# Bimodal spectroscopy integrating multi-wavelength time-resolved photoacoustic spectroscopy and near-infrared spectroscopy with deep learning for quantitative detection of serum biochemical indicators

**DOI:** 10.1016/j.pacs.2026.100858

**Published:** 2026-07-10

**Authors:** Zhong Ren, Chaojun Chen, Gaoqiang Liang, Haibin Zhang, Weinan Shi, Jia Zhang, Guohui Xiao, Xiaoyu Zhu, Wenyan Nie

**Affiliations:** a Key Laboratory of Advanced Electronic Materials and Devices of Jiangxi Province, Jiangxi Science and Technology Normal University, Nanchang, Jiangxi 330038, China; b Key Laboratory of Optic-electronic Detection and Information Processing of Nanchang City, Jiangxi Science and Technology Normal University, Nanchang, Jiangxi 330038, China; c Jiangxi Province Key Laboratory of Immunology and Inflammation, Jiangxi Provincial Clinical Research Center for Laboratory Medicine, Department of Clinical Laboratory, The Second Affiliated Hospital, Jiangxi Medical College, Nanchang University, Nanchang, Jiangxi 330038, China

**Keywords:** Near-infrared spectroscopy, Photoacoustic spectroscopy, SBIs, Multimodal feature fusion, deep learning

## Abstract

To achieve accurate and high-efficient quantitative detection of three serum biochemical indicators (SBIs), namely glucose (GLU), triglycerides (TG), and total cholesterol (TC), this study proposes a bimodal spectroscopy method integrating multi-wavelength time-resolved photoacoustic spectroscopy (TR-PAS) with near-infrared spectroscopy (NIRS), combined with a deep learning (DL) approach. A total of 35 characteristic wavelengths (12 for GLU, 13 for TG, and 10 for TC) are determined from the energy-corrected photoacoustic (PA) peak-to-peak value spectra of biochemical standard solutions by integrating three wavelength selection methods. NIR spectra and time-resolved PA signals at the selected wavelengths are experimentally collected from 1084 serum samples. Following data augmentation for the bimodal data and data preprocessing for NIR spectra, a dual-branch DL network, namely DB-CNN-LSTM-MAM model, was established to quantitatively predict GLU, TG, and TC, respectively. Through optimization of the model structure and parameters, the root mean square error of prediction (RMSEP) and coefficient of determination (R_p_²) for the testing set were as follows: GLU: 0.9653 mmol/L, 0.9296; TG: 0.3956 mmol/L, 0.9575; TC: 0.4443 mmol/L, 0.9290. The generalization of the established DL model is verified by using 10-fold cross validation, and the external independent samples. The quantitiative performances of three SBIs are compared with other DL models. To validate the effectiveness of the proposed method, the comparisons are also performed between the bimodal spectroscopy and unimodal spectroscopy, as well as between the multi-wavelength TR-PAS–NIRS and the single-wavelength TR-PAS–NIRS. The study results indicate that the proposed method provides an accurate and highly efficient candidate solution for the quantitative detection of SBIs of ex vivo serum.

## Graphical Abstract

**Figure:**
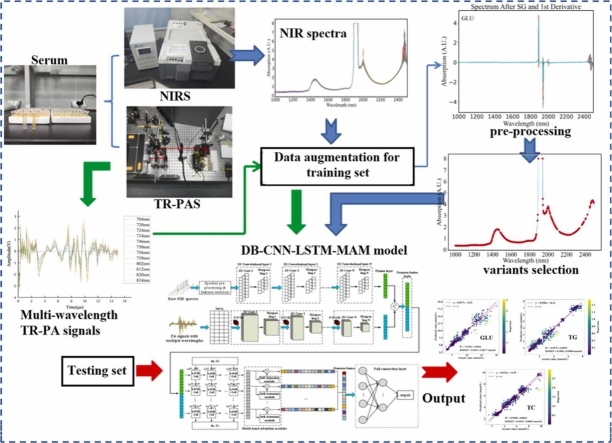

## 1 Introduction

With socioeconomic development, people's dietary standards have continuously improved, and their lifestyles have also undergone significant changes. However, the incidence of various chronic noncommunicable diseases, including cardiovascular disease and the "three-high" diseases (hyperglycemia, hyperlipidemia, and hypertension), has been steadily increasing year by year [1], [2]. It was reported that the number of people suffering from the aforementioned metabolic diseases had exceeded 1.0 billion worldwide by 2021 [3]. These diseases not only significantly affect individuals' quality of life but may also lead to other complications, such as atherosclerosis, coronary heart disease, microvascular complications, retinopathy, nephropathy, and neuropathy [4], [5]. According to reports, the annual number of deaths caused by complications related to the "three-high" diseases is as high as 7 million [6]. Currently, the "three-high" diseases pose a serious threat to global human health, and the situation remains critical. Unfortunately, there is still no effective cure for these conditions. Existing treatment approaches remain primarily prevention-oriented, with supplementary therapeutic interventions guided by the values of serum biochemical indicators (SBIs). Therefore, accurate determination of SBIs associated with these metabolic diseases is of paramount importance.

Conventional clinical approaches for measuring SBIs primarily include biochemical analysis [7], oxidase-based methods [8], [9], electrochemical sensing [10], etc. Biochemical analysis typically requires blood sampling and serum separation via centrifugation, followed by measurement of SBIs levels using biochemical analyzers. Oxidase-based methods, such as the glucose oxidase method [11], rely on the reaction between glucose and oxidase to generate hydrogen peroxide, which is subsequently detected through colorimetry or electrode-based techniques to determine blood glucose concentration. Electrochemical sensing directly reflects SBIs levels by monitoring changes in electrode current or potential. Although these methods yield relatively accurate results, their requirements for blood sampling, complex sample pretreatment, and relatively cumbersome procedures make them less suitable for long-term continuous monitoring. As for minimally invasive methods, while they can reduce physical and psychological discomfort, the risk of secondary infection persists, particularly when frequent SBIs monitoring is required.

In contrast, the efficient and fast approaches exhibit substantial potential for application and can be broadly categorized into optical and non-optical methods. Optical methods include near-infrared spectroscopy (NIRS) [12], [13], mid-infrared spectroscopy [14], Raman spectroscopy [15], and photoacoustic spectroscopy [16], while non-optical methods encompass nuclear magnetic resonance technology [17] and microwave sensing [18]. To date, various studies have investigated SBIs using optical methods. For instance, Wang et al. [19] employed Raman spectroscopy to achieve highly sensitive detection of GLU, TG, and TC. Neves et al. [20] used diffuse reflectance NIRS combined with a partial least squares regression (PLSR) model to determine the levels of GLU, TG, and high-density lipoprotein cholesterol (HDL-C) in rat plasma, achieving root-mean-square error of prediction (RMSEP) values of 6.08, 16.07, and 2.03 mg/dL, respectively. Guaita et al. [21] employed NIRS combined with a PLSR model to detect total protein (TP) in human serum and investigated how predictive performance is influenced by SBIs concentrations. Veettil et al. [22] utilized multimodal spectroscopy, integrating NIRS with attenuated total reflection Fourier-transform infrared spectroscopy (ATR-FTIR), for serum glycine detection; the established PLSR model achieved an RMSEP of 0.303 mg/mL, an R² of 0.999, and a limit of detection of 0.17 mg/mL. Tan et al. [23] employed visible–near-infrared (Vis-NIR) spectroscopy combined with a PLSR model to rapidly and simultaneously analyze direct and indirect bilirubin indicators in serum. Ren et al. [24] utilized UV-Vis-NIR spectroscopy integrated with a deep neural network (DNN) to achieve rapid quantitative detection of GLU, TG, TC, TP, and albumin in human serum. Guleken et al. [25] employed Fourier-transform infrared (FT-IR) spectroscopy combined with principal component analysis (PCA) to distinguish between obese and control groups by detecting obesity-related biochemical changes, including TG and HDL-C levels in human serum. Although the aforementioned optical spectroscopy methods offer advantages such as high efficiency, convenience, and multi-parameter detection capabilities [26], [27], and have demonstrated promising experimental performance, they still face several limitations in practice. These include spectral overlap among multiple components, interruption due to tissue light scattering, strong interference from water absorption, and low sensitivity toward low-concentration analytes [28].

Photoacoustic spectroscopy (PAS) is a hybrid detection technique based on the photoinduced acoustic effect, combining the advantages of high optical resolution and high ultrasonic contrast. In addition, PAS offers high sensitivity, greater penetration depth, compatibility with various light sources, and flexible detection modes [29]. Notably, PAS effectively eliminates interference from light scattering in biological tissues because it detects ultrasonic waves rather than photons. To date, several studies have employed PAS for the analysis of human blood biomarkers. Jansen et al. [30] used intravascular photoacoustic (PA) technology to measure PA signals of cholesterol and its derivatives at 1210 nm. Gazali et al. [31] combined PAS with UV-Vis spectroscopy to investigate the molecular composition of gallbladder stones, confirming that six of the 17 absorption wavebands observed in the PA spectrum (i.e., 330, 390, 500, 590, 640, and 700 nm) corresponded to cholesterol. In addition, PAS has also been frequently used for blood glucose level detection. Tanaka et al. [32], [33] employed resonant PAS and differential continuous-wave PAS to detect *in vivo* blood glucose during oral glucose tolerance tests, achieving standard errors of 64 mg/L and 48 mg/L, and correlation coefficients of 0.84 and 0.80, respectively. Aloraynan et al. [34] improved the signal-to-noise ratio using a PAS method that combines a single-wavelength laser with an absorber plate and a pressure sensor, achieving blood glucose detection with a sensitivity of ±25 mg/dL within the range of 75–300 mg/dL. Maeno et al. [35] obtained a linear correlation between PA spectra and ATR-FTIR spectra from living tissue using a micro-PA cell, achieving a blood glucose prediction accuracy of 70.8%.

Despite its numerous advantages, the PAS method still faces some limitations, primarily due to the constraints of laser light sources and the biological background complexity associated with in vivo measurements. Current research has largely focused on solution mimics, in vitro tissues, or single biochemical markers. Coupled with the limited detection bandwidth of PA sensors, this makes it challenging to effectively identify the characteristic absorption wavelengths of SBIs. Consequently, the application of PAS to explore other SBIs remains significantly hindered [36], [37], [38], [39], [40], [41]. Furthermore, in conventional PAS, PA value spectra are typically obtained by manually scanning wavelengths across a certain waveband at fixed intervals. Then, similar to NIRS, characteristic wavelengths are selected from the preprocessed PA value spectra, followed by the construction of a quantitative model based on the selected wavelengths. The detection efficiency of this conventional PAS method is very low due to its time-consuming nature. In contrast, time-resolved photoacoustic spectroscopy (TR-PAS) enables the direct acquisition of time-resolved PA signals from samples under randomly excited wavelengths. Moreover, TR-PAS provides rich sample information through the profiles, amplitudes, and time shifts of the time-resolved signals. Therefore, to overcome the limitations of single-modality methods and the shortcomings of conventional PAS, a dual-modality detection strategy integrating multi-wavelength TR-PAS with NIRS was proposed for the quantitative prediction of GLU, TG, and TC in serum. This strategy not only fully leverages the complementary advantages of NIRS and PAS, but also improves the detection efficiency and accuracy of SBIs.

Moreover, with the rapid advancement of artificial intelligence (AI) across various fields, its advantages have become increasingly evident in biomedical signal processing and multimodal data modeling. In recent years, numerous machine learning (ML) [42], [43] and deep learning (DL) models have been proposed for medical detection applications. Yang et al. [44] proposed an end-to-end model based on recurrent neural networks (RNNs) to predict blood glucose levels by training on historical data and incorporating Gaussian distribution estimation. Sun et al. [45] employed long short-term memory (LSTM) networks and support vector machine regression (SVR) models to predict blood glucose levels in 20 diabetic patients. Their results indicated that the LSTM model performed best within an 11-minute window; however, its predictive capability progressively declined over extended time frames. Muhammad et al. [46] constructed a convolutional neural network (CNN) model using electrocardiogram segment data to systematically classify healthy individuals and patients with coronary heart disease. The proposed neural network model achieved a classification accuracy of 95.11%. Wang [47] introduced a novel approach combining near-infrared spectroscopy with deep belief networks (DBN) and SVR to enhance blood glucose prediction accuracy, achieving a root mean square error (RMSE) of 0.11 mmol/L and an R² value of approximately 99%. Zhang et al. [48] reported a multi-photon band NIR sensor integrated with a shallow dense neural network (SDNN) and personalized medical features, enabling low-cost blood glucose detection. This system achieved an accuracy of 97.8%, precision of 96.0%, sensitivity of 94.8%, and specificity of 98.7%. Kim et al. [49] constructed a low-density lipoprotein cholesterol (LDL-C) estimation model based on DNN using TC, HDL-C, and TG as inputs. Their results demonstrated significantly superior predictive accuracy on an independent test set compared to the traditional Friedewald equation, highlighting the advantages of DL models in modeling complex nonlinear relationships.

The highlights of this study are presented as follows: First, a dual-modal spectroscopy integrating NIRS and multi-wavelength TR-PAS with DL method is proposed to determinate GLU, TG, and TC. Second, A total of 35 characteristic wavelengths for GLU, TG, and TC are optimally selected via Competitive Adaptive Reweighted Sampling (CARS), Bootstrap Soft Shrinkage (BOSS) and Successive Projections Algorithm (SPA) algorithms. Third, a dual-branch DL model integrating CNN, LSTM networks, and a multi-head attention mechanism (MAM), namely, DB-CNN-LSTM-MAM model, is constructed to quantitatively predict GLU, TG, and TC, respectively. The quantitative prediction performance of the established DL model is validated by comparison with other DL models. Fourth, the effectiveness of biomodal spectroscopy of multi-wavelength TR-PAS–NIRS combined with DB-CNN-LSTM-MAM model was validated through comparison with single-modal spectroscopy and single-wavelength TR-PAS–NIRS.

## 2 Methods and materials

### 2.1 Experimental system

In this study, NIR spectra of serum samples were acquired using a Fourier-transform near-infrared (FT-NIR) spectrometer (Antaris II, Thermofish Co., USA) [24]. The spectral range was from 1000 to 2500 nm with a resolution of 1.2 nm. During the experimental process, the environmental conditions were maintained at a room temperature of 23 ± 0.5 °C and a relative humidity of 35 ± 0.5%. In the NIR experiments, the serum samples were loaded in a high-transmittance quartz cuvette with an optical path length of 2 mm.

The PA signals of serum samples were acquired using a modified PA detection system established in our previous research [50]. In this PA system, a tunable optical parametric oscillator (OPO) pulsed laser (INNOLAS, SpitLight400, Germany) was utilized as the excitation source, with a pulse duration of 7 ns and a repetition frequency of 20 Hz. To mimic the blood flow within human blood vessels, a rubber tube with an inner diameter of 2 mm and a wall thickness of 0.5 mm was employed, and the serum was circulated by a mini water pump. To avoid the impact of chromatic aberration on the PA signals of serum samples during the wavelength tuning of OPO pulsed laser, a reflective objective (LMM15X-P01, Thorlabs Co., USA) with numerical aperture (NA) of 0.3, and working distance of 23.8 mm was utilized to focus the collimated beam into the serum samples in the rubber tube. A linear-focused ultrasonic transducer (2.5C14SJ50XJ, Doppler Co., China) with a center response frequency of 2.75 MHz was used to capture the PA signals. Subsequently, the captured PA signals were amplified by a signal amplifier (ATA−5620, Aigtek Co., China) and then passed through a low-pass filter (BLP−75 +, Mini-Circuits Co., USA) to eliminate high-frequency noise. Finally, the processed data were acquired by a data acquisition card (PCIE−1840, Advantech Co., Taiwan) and transferred to a computer. In experiments, in order to avoid the impact of energy attenuation dependency on excitation wavelength of pulsed laser on the PA signals, during the construction of the blood glucose PA detection system, a beam splitter (BS1419-B, LBTEK Co., China) was incorporated before the reflective objective, which divided the collimated laser beam into two paths. One path was focused by the reflective objective and then incidented into the serum sample to generate PA signals, while the other path was used to monitor the energy of each pulse in real time with an energy meter (PM100D2, Thorlabs Co., USA). Each time the PA signals were collected, the corresponding pulse energy value was also recorded simultaneously. To guarantee the availability and reliability of the experimental data, all samples were subjected to three rounds of collection for NIR spectra and PA data, and their average values were calculated. At the same time, the PA system correction was performed before the PA signal acquisition of all samples to ensure the stability of the PA detection system.

### 2.2 Serum samples and biochemical standard solutions

In the experiments of NIRS and PAS, a total of 1084 human serum samples were utilized. All the serum samples and the true values of SBIs were provided by the Second Affiliated Hospital of Nanchang University. The true values of GLU, TG, and TC in human serum samples were measured using an automatic biochemical analyzer (TBA-FX8, Canon Co., Japan). The reagent lot numbers were as follows: GLU (40719C11), TG (40924E19), and TC (40731B11). These reagents were purchased from Autobio Co., China, between November and December 2024. The quality control (QC) materials, with lot numbers of level 1 (1589UN) and level 2 (1264UE), were purchased from RANDOX Co., UK., also between November and December 2024. For sample collection, venous blood was collected from patients under fasting conditions. After coagulation, the blood was centrifuged using a medical centrifuge at a centrifugal force of 2352 g to separate the serum. The separated serum was stored at 4 °C until examination of SBIs. Approximately 60 min before the experiments, the refrigerated serum samples were removed from storage and allowed to equilibrate to room temperature. Following the acquisition of NIR spectra and PA signals, the serum samples were no longer kept in cold storage and were instead recycled and disposed of by the hospital. The exclusion criteria for measuring SBIs using the biochemical analyzer typically include the following two points: (1) samples with a blood volume of less than 3 mL; and (2) samples with measurement interference caused by conditions such as jaundice, lipemia, or hemolysis.

All human serum samples were the residual serum samples following the patient's diagnosis. Moreover, only one serum sample was provided from one individual, and no multiple serum samples were derived from the same individual. More importantly, a serum sample was only used once in the spectroscopic experiments. Therefore, the patient independence of serum samples was fully guaranteed in this study. The spectroscopic experiments were conducted at Jiangxi Science and Technology Normal University (JXSTNU) and were approved by the Ethics Committee of JXSTNU (No.: IRBJXSTNU−2024009). The Analysis of Variance (ANOVA) for GLU, TG, and TC of the human serum samples, conducted via Origin 2025, is presented in Table 1.

**Table 1:** Statistic information of serum samples.

SBIs	Maximum	Minimum	Mean	SD^1^	MAD^2^	CV^3^（%）
GLU	17.7	2.04	6.17	1.82	1.17	29.45
TG	16.22	0.31	1.68	1.33	0.82	79.38
TC	10.65	1.57	4.88	1.18	0.93	24.22

SD^1^: standard deviation; MAD^2^: mean absolute deviation, CV^3^: coefficient of variation.

To optimally select the characteristic wavelengths of GLU, TG, and TC in serum, pure GLU, TG, and TC standards purchased from Hefei Jinnu Biotechnology Co., Ltd, China, were used in the PA experiments. The purities of the GLU, TG, and TC standards are GLU≥ 99%, TG≥ 98%, and TC≥ 96%, respectively. For GLU, ultrapure water was employed to prepare GLU standard solutions with varying concentrations. Given that TG and TC are water-insoluble lipid substances, anhydrous ethanol (Bolinda Co., China) was used as a solvent to prepare TG and TC standard solutions of different concentrations. In this work, four GLU standards solution were prepared using 0.1 L pure water, respectively, i.e., 151.2 mg/dL,158.4 mg/dL, 190.8 mg/dL, 207.0 mg/dL. Five TG standards solution were prepared using 0.1 L anhydrous ethanol, respectively, i.e., 49.62 mg/dL, 149.73 mg/dL, 249.85 mg/dL, 349.97 mg/dL, 450.09 mg/dL. Five TC standards solution were also prepared using 0.1 L anhydrous ethanol, respectively, i.e., 49.88 mg/dL, 150.04 mg/dL, 249.81 mg/dL, 349.96 mg/dL, 450.12 mg/dL. To minimize the significant change in the concentration of TG and TC solutions caused by ethanol volatilization, the TG and TC standard solutions were placed in a sealed mini-plastic container, and the temperature was maintained at approximately 23 ± 0.5 °C during the experiments.

### 2.3 CNN

Generally, CNNs are utilized for processing two-dimensional data, especially in the field of image processing [51], [52]. In contrast, one-dimensional CNN (1DCNN) models are primarily employed for dealing with one-dimensional data, such as time-series data or one-dimensional signals [53], including audio signals, stock data, spectral data, etc.

**Fig. 1:**
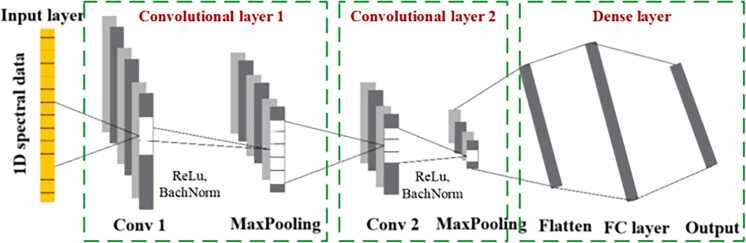
The basic architecture diagram of 1DCNN model.

The general architecture of a 1DCNN with two convolutional layers is depicted in Fig. 1. One- dimensional spectral data are input into the input layer. Subsequently, convolutional layers and pooling layers are sequentially designed to extract features. In the convolutional layers, a series of convolutional kernels with similar or different sizes at different levels are employed. Then, an activation function, such as the Rectified Linear Unit (ReLU) [54] or Parametric Rectified Linear Unit (PReLU) [55], is used to conduct the nonlinear transformation of the extracted features. In the pooling layers, the maximum or average pooling methods are applied for feature dimensionality reduction. Then, the extracted features are reshaped into a one-dimensional vector through the flatten layer. Finally, a fully connected layer and an output layer are used to perform the regression task.

### 2.4 LSTM

Long Short-Term Memory (LSTM) is a variant model of the Recurrent Neural Network (RNN). It addresses the long-distance dependency issue of time-series data and alleviates the problems of gradient vanishing and exploding [56], [57], [58]. Moreover, LSTM possesses a more powerful memory capacity and exhibits greater robustness against noise and redundant inputs [59].

**Fig. 2:**
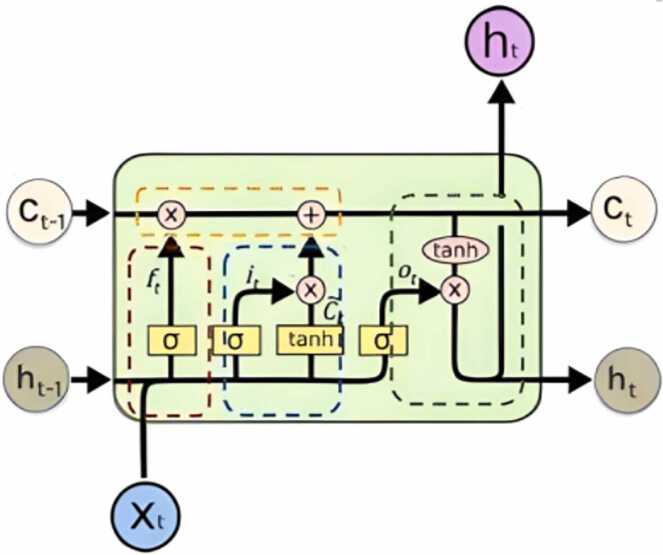
LSTM internal cell structure.

Fig. 2 depicts the internal structural unit of LSTM, which encompasses an input gate, $i^{\left(t\right)}$a forgetting gate$f^{\left(t\right)}$, an output gate$o^{\left(t\right)}$, and an internal memory unit$C^{\left(t\right)}$.

Regarding the input gate$i^{\left(t\right)}$, which determines the amount of new information ${\tilde{C}}^{\left(t\right)}$required to be added to the cell state at the current moment:

(1) $$i^{\left(t\right)}=\sigma \left(W_{i}\cdot \left[h^{\left(t-1\right)},x^{\left(t\right)}\right]+b_{i}\right)$$

(2) $${\tilde{C}}^{\left(t\right)}=\;\tanh(W_{c}\cdot [h^{\left(t-1\right)},x^{\left(t\right)}]+b_{f})$$.

Concerning the forget gates$f^{\left(t\right)}$, which determine the extent to which the previous cellular state$C^{\left(t-1\right)}$ needs to be retained:

(3) $$f^{\left(t\right)}=\sigma (W_{f}\cdot [h^{\left(t-1\right)},x^{\left(t\right)}]+b_{f})$$.

The output gate $o^{\left(t\right)}$and the final hidden state are presented as follows:

(4) $$o^{\left(t\right)}=\sigma (W_{o}\cdot [h^{\left(t-1\right)},x^{\left(t\right)}]+b_{o})$$

(5) $$h^{\left(t\right)}=o^{\left(t\right)}⊗\;\tanh(C^{\left(t\right)})$$.

For the cell state update$C^{\left(t\right)}$, which synthesizes the information from the input gate and the forget gate to calculate the new cell state, namely,

(6) $$C^{\left(t\right)}=f^{\left(t\right)}⊗C^{\left(t-1\right)}+i^{\left(t\right)}⊗{\tilde{C}}^{\left(t\right)}$$.

In (1), (3), (4), σ(⋅)is the Sigmoid activation function constrained between (0, 1), which indicates the degree of opening and closing of the control gate. tanh(⋅) is the hyperbolic tangent function, which is employed to non - linearly transform the cell state so that it lies within the range of (−1, 1). $W_{i\;},W_{f}\;,W_{o}\;,W_{c}$is the weight matrix that determines the influence of the input and hidden states on the gated cell. $b_{i}\;,b_{f}\;,b_{o}\;,b_{c}$are the bias terms, which are utilized to adjust the output of the activation function. ⊙represents the element-by-element multiplication for selective messaging of the gating.

### 2.5 MAM module

Traditional RNNs or CNNs can only concentrate on local or short-term dependencies when handling sequence data. In contrast, attention mechanism (AM) module enables each element in the sequence to “focus” on the entire sequence and assigns different weights based on its relevance, thereby capturing the effectiveness of long-distance dependencies [60]. The MAM is an enhancement of the attention mechanism. Its core concept is to project the input into multiple sub-spaces, independently compute the attention, and then integrate the results to capture diverse features [61], [62], [63]. The calculation process of the MAM is presented as follows:

(1) Input projection
  Let the input sequence be $X\in R^{n\times d}$, independent queries (***Q***), keys (***K***) for each head ***h*** (total ***H*** heads),value (***V***) projection matrices are generated:
  (7) $$Q_{h}=XW_{h}^{Q},K_{h}=XW_{h}^{K},\;V_{h}=XW_{h}^{V}$$
  where $W_{h}^{Q},W_{h}^{K}\in R^{d\times d_{k}},\;W_{h}^{V}\in R^{d\times d_{v}}$, generally$d_{k}=d_{v}=d/H$.
(2) Single-head attention calculation
  For each head ***h***, the scaled dot product attention is computed, i.e.,
  (8) $$\mathrm{Attention}(Q_{h},K_{h},V_{h})=\mathrm{softmax}\left(\frac{Q_{h}K_{h}^{T}}{\sqrt{d_{k}}}\right)V_{h}$$
  where $\frac{Q_{h}K_{h}^{T}}{\sqrt{d_{k}}}$ is the score matrix, which measures the similarity of the query and key. After softmax normalization, the attention weights are obtained, and the value matrix $V_{h}$ is weighted and summed to obtain the output of the head ***h***,$O_{h}\in R^{n\times d_{v}}$.
(3) Merging of multiple outputs.

The outputs of all heads are concatenated and the dimensions are adjusted through linear projection:

(9) $$\mathrm{MultiHead}(X)=OW^{O}$$.

The final output is consistent with the input dimension ***d***.

## 3 Experimental results

### 3.1 PA experimental results for biochemical standard solutions

**Fig. 3:**
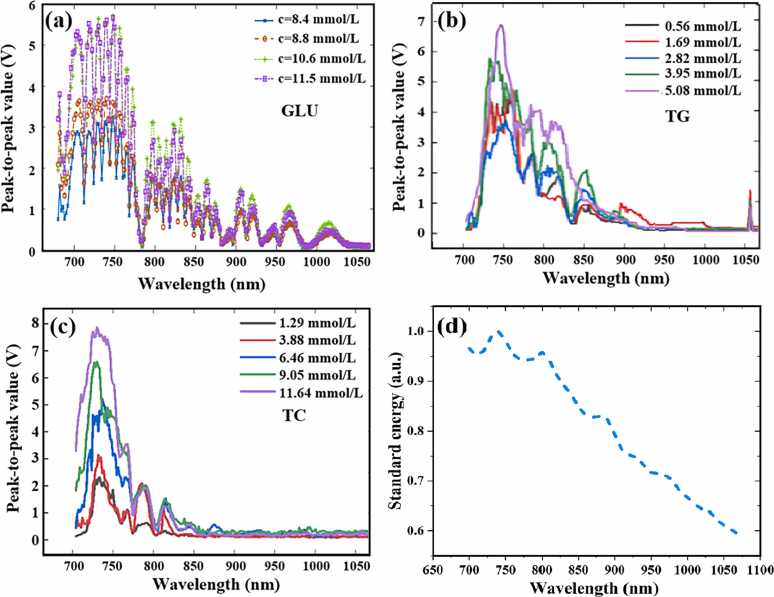
Parts of energy-corrected PA peak-to-peak value spectra of GLU (a), TG (b), and TC (c) standard solutions; the standarded output energies of pulsed laser (d).

The PA peak-to-peak value spectra of GLU, TG, and TC standard solutions at various concentrations were acquired within the wavelength range of 700–1070 nm with a 2 nm interval. To overcome the impact the energy attenuation dependent to the excitation wavelengths of pulsed laser on the PA peak-to-peak values, dividing the acquired PA peak-to-peak values of serum samples at each wavelength by the energy values recorded via the energy meter during experiments, the energy-corrected PA peak-to-peak values were obtained, which are depicted in Fig. 3(a)-(c), respectively. The standarded output energies of pulsed laser from 700 nm to 1070 nm are presented in Fig. 3(d).

Fig. 3(a)-(c) illustrate that the PA peak-to-peak value spectra of the aforementioned three standard solutions exhibit several distinct peaks within the wavelength range of 700–850 nm. This phenomenon indicates that the light absorption within this wavelength band is relatively stronger. Beyond 850 nm, the PA signals gradually decay and tend towards lower stable values, indicating that the light absorption in this wavelength band is weak and the PA effect is diminished. Additionally, the variations in the PA peak-to-peak values of the three standard solutions at different concentrations demonstrate that the overall PA intensities increase with the concentration, but the degree of enhancement varies according to the substances.

### 3.2 Results of characteristic wavelengths selection for SBIs

**Fig. 4:**
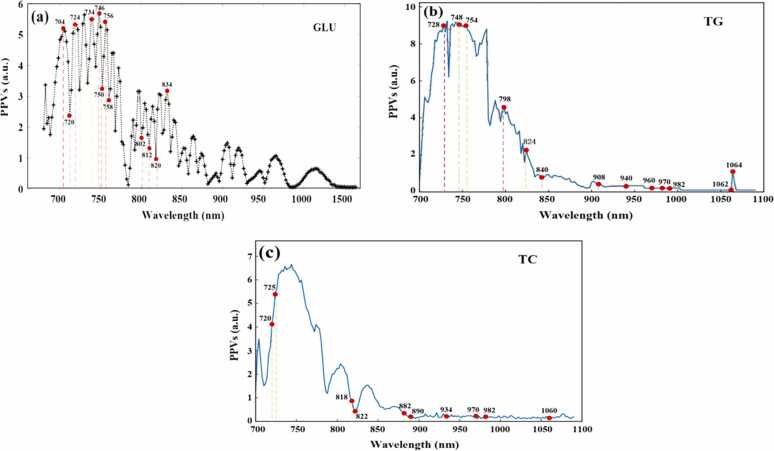
The selected characteristic wavelengths of GLU (a), TG (b), and TC (c).

As we know, for the collected PA peak-to-peak value spectra of serum samples, it not only contains plenty of unuseful information, which will seriously impact the quantitative prediction performance of BSIs, but also too much wavelengths TR-PA signals will make the structure of model complicated, as well as huge volume of parameters, which will prolong the training time of model, even impact the prediction performance. Therefore, the characteristic wavelengths optimal selection is necessary for the quantitative modeling of multi-wavelength TR-PA signals. In the past, the single method of characteristic wavelengths optimal selection was usually utilized. However, the selected characteristic wavelengths are usually too much, even hundreds of wavelengths were selected, that is, some useless variants may be selected as the characteristic wavelengths. In the selection of optimal characteristic wavelengths, the CARS method is widely used as a classic approach due to its strong ability to determine characteristic wavelengths, effectively optimize model performance, and enhance prediction accuracy. However, due to the high collinearity of spectra, some "weak information" variables with small coefficients in the model may contribute significantly when combined with other variables. CARS tends to directly eliminate such low-coefficient variables in one iteration, leading to information loss and overfitting. To overcome this limitation of the CARS algorithm, three optimal selection algorithms, namely CARS [64], BOSS [65], and SPA [66], were utilized for the PA peak-to-peak value spectra to obtain the optimal characteristic wavelengths of three SBIs in this study. Among them, the "soft shrinkage" strategy of BOSS is specifically designed to address this issue. Instead of directly eliminating variables, it continuously gives all variables (including those variants with low coefficients) the opportunity to be selected through weighted sampling. This makes the wavelengths selected by BOSS closer to the theoretical absorption bands, effectively eliminating interference while fully retaining key information, thereby constructing a model with stronger predictive ability and lower risk of overfitting. Additionally, SPA can perform extreme compression on spectral variables through a forward selection process that minimizes vector collinearity. Through projection analysis, it selects the few key variables with the lowest information redundancy from hundreds or thousands of wavelengths, constructing a minimalist model that effectively avoids collinearity interference, has a simpler structure, and greatly enhances robustness. The characteristic wavelengths selected for GLU, TG, and TC are presented in Fig. 4(a)-(c), respectively. In Fig. 4, the selected characteristic wavelengths are marked with red dots.

From Fig. 4(a)-(c), it can be observed that the characteristic wavelengths are primarily located at the peaks and troughs of the PA peak-to-peak value spectra of GLU, TG, and TC standard solutions. The selected characteristic wavelengths of GLU are 704, 720, 724, 734, 746, 750, 756, 758, 802, 812, 820, and 834 nm, respectively. The selected characteristic wavelengths of TG are 728, 748, 754, 798, 824, 840, 908, 940, 960, 970, 982, 1062, and 1064 nm, respectively. The selected characteristic wavelengths of TC are 720, 725, 818, 822, 882, 890, 934, 970, 982, and 1060 nm, respectively.

### 3.3 Experimental results of NIRS and multi-wavelength TR-PAS

**Fig. 5:**
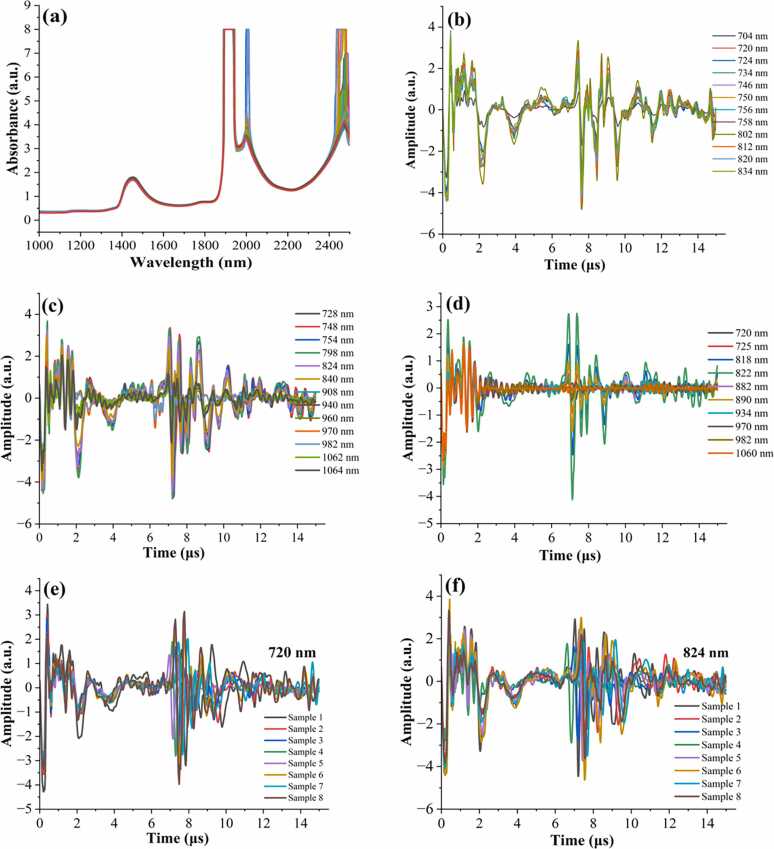
NIR spectra (a), and time-resolved PA signals for selected human serum samples at multiple characteristic wavelengths of GLU (b), TG (c), and TC (d); the time-resolved PA signals of serum samples at characteristic wavelengths of 720 nm (e) and 824 nm (f).

The NIR spectra of serum samples were acquired using a FT-NIR spectrometer within the wavelength range of 1000–2500 nm. The NIR spectra of some serum samples are shown in Fig. 5(a).

As can be observed from Fig. 5(a), although the NIR spectral profiles of different serum samples exhibit similarities within the 1000–2500 nm range, there are disparities in absorbance values. The difference in absorbance may be attributed to the concentrations of SBIs, such as GLU, TG, and TC. From Fig. 5(a), apart from the characteristic wavelengths of water molecules near 1450 nm and 1930 nm, characteristic wavelengths for GLU, TG, and TC can also be identified. For GLU, the characteristic wavelengths are 1200 nm, 1300 nm, 1790 nm, and 2000 nm [67], [68]. For TG, the characteristic wavelengths are 1200 nm, 1400 nm, 1600–1800 nm, 2100–2200 nm, and 2300–2350 nm [69]. For TC, the characteristic wavelengths are 1650–1730 nm and 1700–1798 nm [70], which are due to the steroidal ring skeleton in the molecular structure of TC and the methyl (-CH_3_) and methylene (-CH_2_-) groups on the alkyl side-chains. Therefore, the characteristic absorption region of GLU overlaps with those of TG and TC (e.g., around 1700 nm), presenting challenges for the quantitative prediction and differentiation of SBIs. To solve this issue, NIRS combined with multi-wavelength TR-PAS and DL method was proposed to achieve high-accuracy quantitative prediction of GLU, TG, and TC.

To achieve this aim, the time-resolved PA signals of all serum samples were collected. The energy-corrected time-resolved PA signals of a portion of serum samples at the selected characteristic wavelengths of GLU, TG, and TC are obtained and presented in Fig. 5(b)-(d), respectively. From Fig. 5(b)-(d), it is observable that the maximum amplitudes of the time-resolved PA signals of serum samples are situated at approximately 7.0 μs. Based on the diameters of the imitation vessel and the distance between the ultrasonic transducer and the imitation vessel, it can be deduced that the focused laser spot falls within the cavity of the imitation vessel. The maximum amplitude corresponds to the intensity response of serum samples at the PA excitation source. As depicted in Fig. 5(b)-(f), within the time range of 0–4 μs, there exists the initial excitation signal of the ultrasonic transducer. In the time range of 12–15 μs, there are the clutter signals of serum samples in the cavity of the imitation vessel. Simultaneously, from Fig. 5(b)-(d), it can be noted that the profiles of the time-resolved PA signals of a serum sample at the different characteristic wavelengths of GLU, TG, and TC are similar, yet the amplitudes differ. This is because the optical absorptions of a serum sample with fixed physical properties vary at different wavelengths. Fig. 5(e) and (f) respectively present the time-resolved PA signals of 8 serum samples at two characteristic wavelengths of 720 nm and 824 nm. From Fig. 5(e) and (f), it can be observed that the profile, amplitude, and peak shift of the time-resolved PA signals for different serum samples all vary at the same excitation wavelength. The reason is that the optical, ultrasonic, and thermal properties of different serum samples are diverse owing to different concentrations of SBIs. Therefore, the alterations in NIR spectra and the time-resolved PA signals of serum samples offer evidence for predicting the concentrations of GLU, TG, and TC by employing the NIRS combined with the multi-wavelength TR-PAS method in this study.

## 4 Quantitative results of SBIs

### 4.1 Data augmentation

**Fig. 6:**
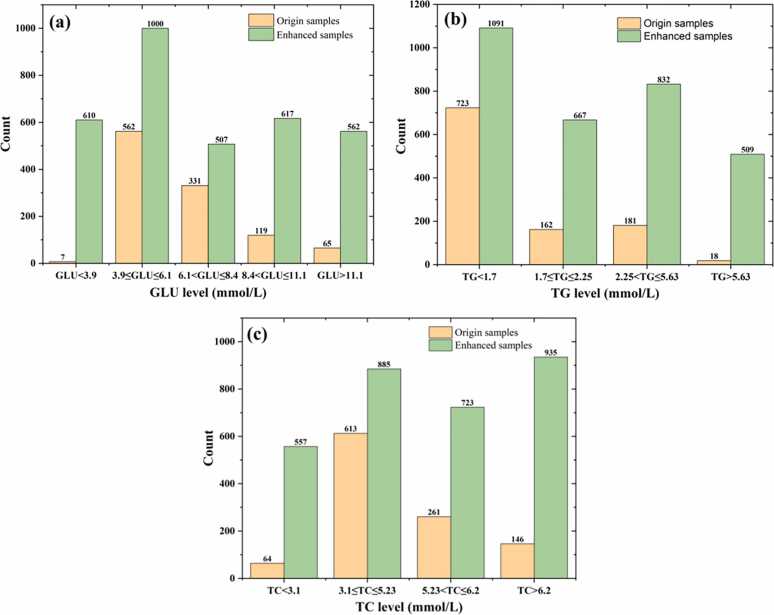
Number distribution histograms of original serum samples and data-augmented serum samples in different level intervals for GLU (a), TG (b), and TC (c).

In this study, despite the acquisition of experimental data from 1084 human serum samples, the issue of sample number imbalance emerged across different levels of SBIs. Fig. 6(a)-(c) present the distribution histograms of serum samples at different level intervals of GLU, TG, and TC. From Fig. 6(a), it is evident that for GLU, the quantity of serum samples at the lower level (GLU < 3.9 mmol/L) and the serious level (GLU > 11.1 mmol/L) is relatively small, while the number of serum samples at the normal level (3.9 ≤ GLU < 6.1 mmol/L) and the slightly higher level (6.1 ≤ GLU < 8.4 mmol/L) is large. Regarding TG, there is a large number of serum samples at the normal level (TG < 1.7 mmol/L), yet the number of serum samples at the serious level (TG > 5.63 mmol/L) is small. For TC, the number of serum samples at the lower level (TC < 3.1 mmol/L) and the serious level (TC > 6.2 mmol/L) is smaller compared to those at the normal level (3.1 ≤ TC ≤ 5.2 mmol/L). This data imbalance problem will cause the quantitative model to generally fail to learn comprehensive features during the training stage, resulting in a poor model fitting effect and weak generalization ability of the model [71], [72]. Moreover, for DL modeling, it typically demands a large number of samples for effective model training. However, in practical applications, obtaining more samples is often restricted by time and cost factors. In this study, to address this problem in the context of insufficient sample size for DL modeling of SBIs quantitative prediction, the data augmentation method based on the Dynamic Time Warping Barycenter Averaging (DTWBA) algorithm [73] was applied within each divided level interval of SBIs.

In order to avoid data leakage, before the data augmentation, 1084 original human serum samples were divided into the training set and the testing set in ratio of 8:2 in each divided level interval of SBIs. Moreover, the data augmentation was performed for the training set. The total number of serum samples including the augmented samples and the original samples in each level interval for GLU, TG, and TC is shown in Fig. 6(a)-(c), respectively. From Fig. 6(a)-(c), it can be observed that the number of serum samples at the low level and serious level of SBIs was significantly increased, and the sample imbalance problem was effectively resolved. After data augmentation, the total numbers of serum samples for predicting GLU, TG, and TC are 3296, 3099, and 3100, respectively.

The parts of data-augmented NIR spectra of human serum samples obtained through the DTWBA algorithm are presented in Fig. S1(a) of the Supplementary Material. Moreover, the data-augmented time-resolved PA signals at multiple characteristic wavelengths of GLU, TG, and TC are respectively provided in Fig. S1(b)-(d) of the Supplementary Material.

In Fig.S1(b)-(d), the time-resolved PA signals collected in the experiment encompass the initial excitation signals and clutter signals that are unrelated to the concentration of SBIs. To mitigate the interference of these non-essential signals on the deep learning model, the initial 200 data points (corresponding to the initial excitation signals) and the final 100 data points (clutter signals) of each time-resolved PA signal were eliminated. Consequently, only the crucial portion of the time-resolved PA signals of human serum samples, consisting of approximately 500 data points, was retained and utilized as the input data for quantitative modeling. From Fig.S1(a)-(d), it is evident that the data-augmented NIR spectra and time-resolved PA signals of serum samples bear resemblance to those of the real NIR spectra and time-resolved PA signals. This similarity not only provides a sufficient data source for the quantitative prediction of GLU, TG, and TC in serum samples based on the DL model but also enhances the generalization ability of the DL model.

### 4.2 NIR spectra pre-processing

**Fig. 7:**
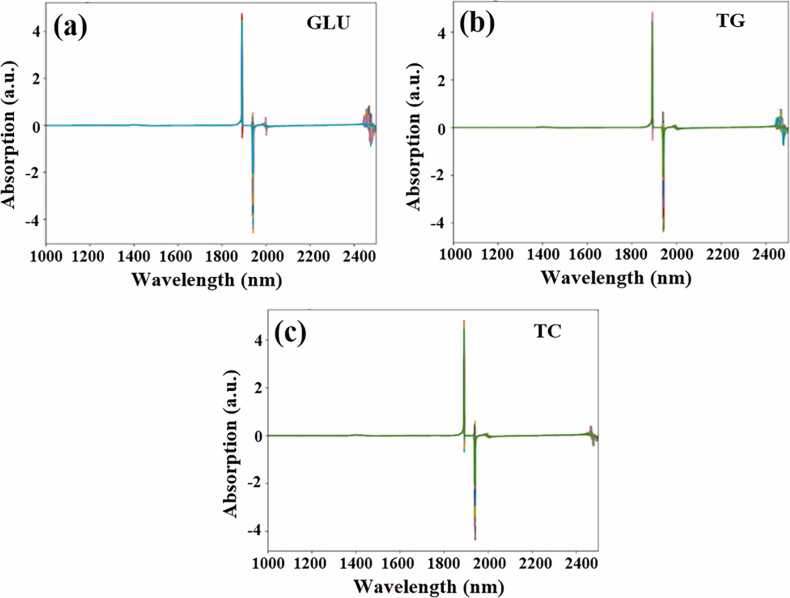
Preprocessed NIR spectra by using SG + FD method for GLU (a), TG (b), and TC (c).

In experiments, the collected NIR spectra of human serum are frequently perturbed by extraneous factors such as sample state, ambient temperature, stray light, and instrument response. These perturbations result in baseline drift and spectral overlap, thereby influencing the prediction accuracy of SBIs through quantitative modeling. To address this issue, several spectral pre-processing algorithms, including First-order Derivative (FD) [74], Savitzky-Golay smoothing (SG) [75], Multiplicative Scatter Correction (MSC) [76], Moving Average Filter (MAF) [77], Standard Normal Variate (SNV) [78], and their combined methods (SG + FD, MSC + FD, MAF + FD, SNV + FD, SNV + SG, and MSC + SG), were employed to pretreat the raw NIR spectra of training set. Furthermore, the optimal pre-processing algorithms for GLU, TG, and TC were determined based on the PLSR model [79]. The performance evaluation indices, including the RMSEC and R_c_^2^ for the calibration set, as well as the RMSEP and R_p_^2^ for the prediction set of GLU, TG, and TC based on different spectral pre-processing algorithms, are presented in Table S1 of the Supplementary Material. From Table S1, it can be observed that the SG + FD method has yielded the most favorable spectral pre-processing results for GLU, TG, and TC, as it exhibits the smallest RMSEC and RMSEP values, along with the largest R_c_^2^ and R_p_^2^ values compared to other methods. Fig. 7(a)-(c) present the NIR spectra of GLU, TG, and TC pre-processed by the SG + FD method, respectively.

### 4.3 Feature variants selection for NIR spectra

**Fig. 8:**
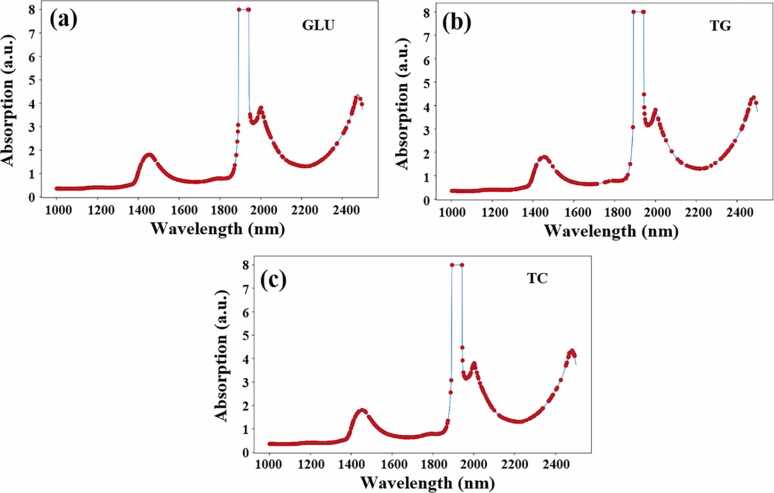
Distribution of selected features of GLU (a), TG (b), and TC (c) via CARS algorithm.

Generally, NIR spectra are not only high-dimensional variable data but also contain a substantial amount of redundant information. This redundancy increases the complexity of the model's structure and parameters and is prone to causing overfitting issues. To address this problem, the CARS algorithm was utilized for feature variable selection of training set’s NIR spectra in this study. The selected variables of NIR spectra for GLU, TG, and TC were marked with red dots in Fig. 8(a)-(c), respectively. The numbers of selected variables for GLU, TG, and TC were 458, 349, and 457, respectively. It can be noted that the majority of these selected variables are located near the peaks and troughs of the spectra, as well as in regions with significant changes. By selecting these key variables, the data dimension, the interference of noise, and redundant information were diminished, which can contribute to enhancing the accuracy and stability of quantitative modeling. Moreover, this targeted feature variable selection facilitates the establishment of robust calibration models even under varying instrumental conditions or sample matrices. By retaining critical variances while suppressing irrelevant fluctuations, this feature selection approach ensures consistent prediction performance across diverse experimental settings. Additionally, the interpretability of the modeling can be improved by highlighting the most relevant spectral variables associated with each SBIs.

### 4.4 Quantitative prediction based on DL model

**Fig. 9:**
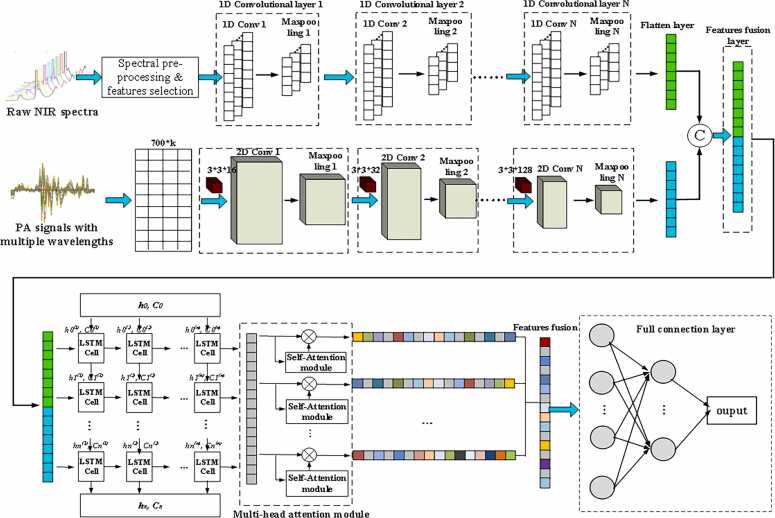
Structure of the DB-CNN-LSTM-MAM model.

To achieve the quantitative prediction of GLU, TG, and TC in human serums, this study established a DNN, specifically a dual-branch convolutional neural network combined with LSTM and MAM modules, namely the DB-CNN-LSTM-MAM model, by leveraging the collected dual-modality data, namely NIR spectra and multi-wavelength time-resolved PA signals. In the NIRS branch, a one-dimensional convolutional neural network (1DCNN) with multiple convolutional layers was employed to extract the significant features from the NIR spectra. In the PAS branch, the multi-wavelength time-resolved PA signals were transformed into a two-dimensional (2D) matrix. Subsequently, a 2DCNN with multiple convolutional layers was utilized to synchronously extract the significant features from the multi-wavelength time-resolved PA signals. The features extracted from the dual branches were fused into a 1D vector using the feature-level fusion strategy. Then, the fused features were successively input into the LSTM module and the MAM module to further extract the long-term dependency features and key features. Finally, a three-layer fully connected network was employed to obtain the predicted values of GLU, TG, and TC, respectively. The structure of the DB-CNN-LSTM-MAM model is presented in Fig. 9.

The structure and parameters of DB-CNN-LSTM-MAM model for predicting GLU, TG, and TC are given in the Table S2 of Supplementary Material.

Based on the established DB-CNN-LSTM-MAM model, five times of repeated training were performed to reduce the contingency of prediction results. To avoid the overfitting of model, L_2_ regularization was utilized in Loss function. The quantitative prediction results and statistic information of training set and testing set for GLU, TG, and TC at five repeated training times based on BD-CNN-LSTM-MAM model are presented in Table S3 of Supplementary Material.

**Fig. 10:**
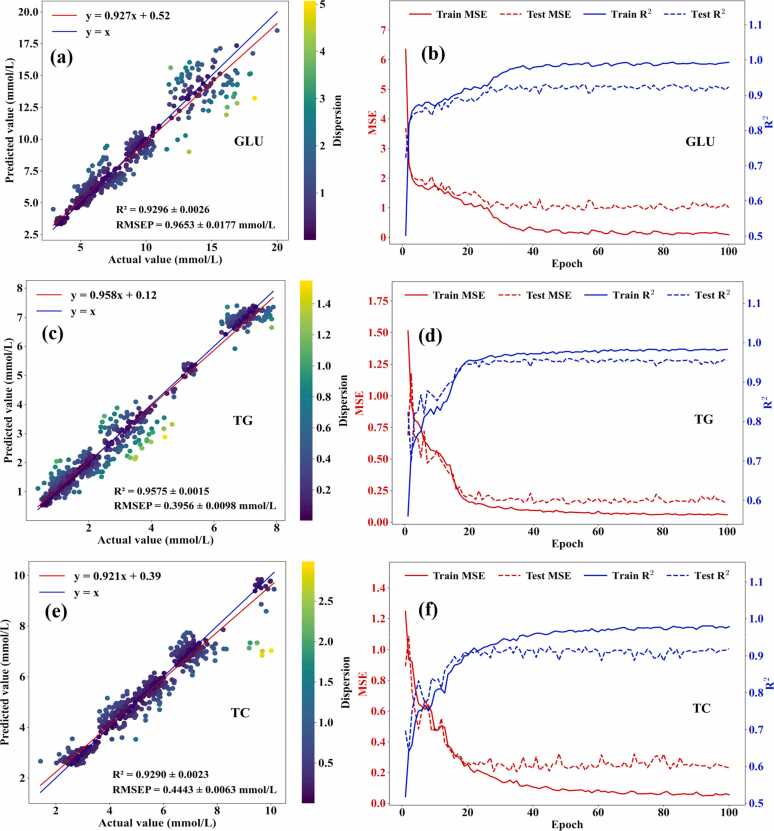
Quantitative average prediction results of GLU (a), TG (c), and TC (e) based on DB-CNN-LSTM-MAM model for the testing set of serum samples; the training curves of training set and testing set for GLU(b), TC(d),and TC(f).

From Table S3, it can be seen that the average RMSEC of GLU, TG, and TC for the training set are 0.376 mmol/L, 0.280 mmol/L, and 0.295 mmol/L, with R_c_^2^ of 0.989, 0.977, and 0.964, respectively. For the testing set, the average RMSEP of GLU, TG, and TC were 0.9653 mmol/L, 0.3956 mmol/L, 0.4443 mmol/L, with R_p_^2^ of 0.9296, 0.9575, and 0.9290, respectively. The average predicted results of GLU, TG, and TC for the testing set based on the DB-CNN-LSTM-MAM model are shown in Fig. 10(a), (c), and (e), respectively. The training curves of training set and testing set for GLU, TG, and TC are presented in Fig. 10(b), (d), and (f), respectively. In Fig. 10(a), (c), and (e), the horizontal coordinates indicate the actual measured values, and the vertical coordinates indicate the predicted values of three SBIs. The colors of the points shown by the bars indicate the dispersion degree of data, with the dark blue color indicating a low degree of dispersion, and the yellow color indicating a larger degree of dispersion. The red line in the figure is the linearly fitted line between the predicted and actual values and is labeled with its slope and intercept. The blue line serves as a reference line, i.e., *y = x*. Moreover, from Fig. 10(b), (d), and (f), it can be seen that although the prediction performances of training set are superior to those of the testing set in the epoch of 100 for GLU, TG, and TC, their deviation is approximately 2–5%. Therefore, from Fig. 10(a)-(f) and Table S3, it can be known that the quantitative prediction model of DB-CNN-LSTM-MAM has not overfitted. In addition, to verify the generalization performance of DB-CNN-LSTM-MAM model, the 10-fold cross validation were performed for training set, the mean and standard deviation (SD) values of RMSE and R^2^ (RMSECV, and R_CV_^2^) for GLU, TG, and TC of training set were obtained, which are presented in Table S3. From Table S3, it can be seen that the mean ± SD values of RMSECV and R_CV_^2^ for GLU are 0.993 ± 0.095 mmol/L and 0.927 ± 0.016, respectively; the mean and standard deviation values of RMSECV and R_CV_^2^ for TG are 0.424 ± 0.02 mmol/L and 0.947 ± 0.013; the mean and standard deviation values of RMSECV and R_CV_^2^ for TC are 0.450 ± 0.048 mmol/L and 0.914 ± 0.019. The R_p_^2^ values of GLU, TG, and TC for testing set all fall into the mean ± SD ranges of R_CV_^2^ for 10-fold cross validation. Therefore, the generalization performance of DB-CNN-LSTM-MAM model is good. Overall, the prediction performances of GLU, TG, and TC based on DB-CNN-LSTM-MAM model is satisfactory.

## 5 Discussion

### 5.1 Ablation experiments and comparison between different CNN models for biomodal spectroscopy

In this work, the selection of the hybrid deep learning framework combining CNN, LSTM and MAM follows the following core rules. First, CNN is selected because it can effectively extract local deep features from the one-dimensional NIR spectral data and multi-wavlengths TR-PA signals, automatically capturing the implicit correlation and characteristic information of different characteristic wavelengths through convolutional operations, which greatly reduces the difficulty of manual feature extraction. Second, considering that NIR spectral data and multi-wavlengths TR-PA signals can be regarded as the sequence of data points arranged by optical energy absorption, LSTM is introduced to capture the sequential dependence between different wavelength variables with long-distance dependency relationship, which can better mine the context information of the sequences and avoid the loss of potential effective information. Finally, the MAM module is integrated to assign different weight coefficients to the extracted features from multiple viewpoints: key features related to the concentrations of target BSIs (GLU, TG, and TC) will obtain higher weights, while useless interference features will be assigned lower weights, which further effectively filters noise and useless features, enhances the model's focus on effective information, and ultimately improves the overall prediction accuracy and generalization ability of the model.

**Fig. 11:**
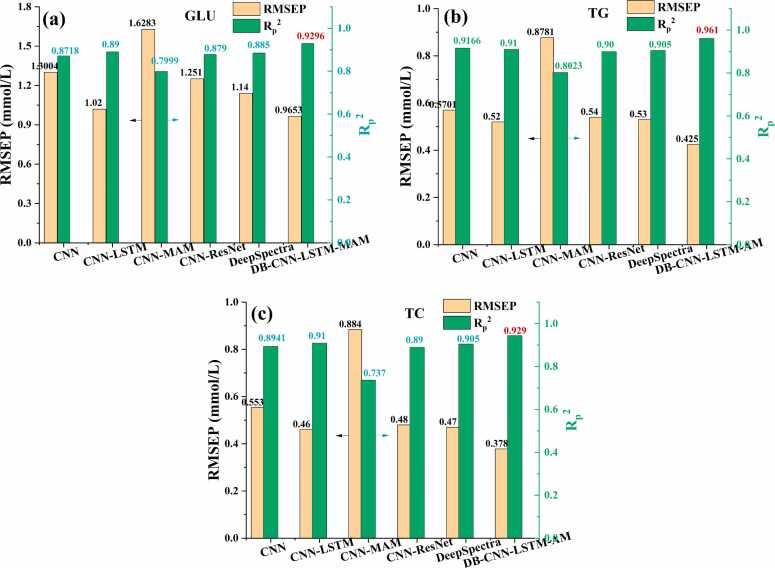
Prediction performance comparison of GLU (a), TG (b), and TC (c) between DB-CNN-LSTM-MAM model and other CNN models, as well as ablation experimental results.

To validate the prediction performance of GLU, TG, and TC based on DB-CNN-LSTM-MAM model, a comparative analysis was conducted between the proposed DL model and three other DL models, i.e., CNN-ResNet model, and DeepSpectra model. For CNN-ResNet model, the biomodal features were extracted via 1DCNN−1DResNet, and 2DCNN−2DResNet modules in dual branches, respectively. After that, the flatten biomodal features were fused via concatenation operation, and directely transferred into fully connection layers. In the case of the DeepSpectra model, the biomodal features were extracted via 1DCNN−1DInception modules, and 2DCNN−2DInception modules, respectively. Then, the flatten biomodal features were fused via concatenation operation, and directely transferred into fully connection layers. By adjusting the model architecture and optimizing parameters, the comparative results (RMSEP, and R_p_^2^) of these models for GLU, TG, and TC of testing set are presented in Fig. 11 (a)-(c), respectively. As can be observed from Fig. 11(a)-(c), the RMSEP values of GLU, TG, and TC in the testing set based on the DB-CNN-LSTM-MAM model are lower than those of the CNN-ResNet model and DeepSpectra model. Moreover, the coefficient of determination (R_p_^2^) values of the DB-CNN-LSTM-MAM model are the highest.

To further verify the influence of different modules on the quantitative performance of DB-CNN-LSTM-MAM model, the ablation experiments of the established model were performed. The models used in the ablation experiments are CNN model, CNN-LSTM model, CNN-MAM model, and DB-CNN-LSTM-MAM model. For CNN model, the LSTM and MAM modules were all deleted from DB-CNN-LSTM-MAM model, that is, the biomodal features for the preprocessed NIR spectra and time-resolved PA signals with multiple characteristic wavelengths were extracted via 1DCNN and 2DCNN modules in dual branches, respectively. Subsequently, the flatten biomodal features were fused via concatenation operation, and transferred into the fully connection layers. For CNN-LSTM model, the MAM module was deleted from DB-CNN-LSTM-MAM model, that is, after the features extracted from NIRS branch and PAS branch with CNN structrue, the flatten biomodal features were fused via concatenation operation, and transferred into LSTM module and fully connection layers. For CNN-MAM model, the LSTM module was deleted from DB-CNN-LSTM-MAM model. For these four aforementioned models, their RMSEP and R_p_^2^ values of testing set for GLU, TG, and TC were obtained, which are presented in Fig. 11 (a)-(c), respectively. From Fig. 11(a)-(c), it can be seen that the performance of CNN model is lower than those of the CNN-ResNet model and DeepSpectra model. The reason may be the 1DCNN−1DResNet, and 2DCNN−2DResNet modules in dual branches of CNN-ResNet model can extract deeper NIR spectral and PA feature representations through residual connections, avoiding the degradation of feature information caused by shallow network structures, so it achieves better prediction results than the simple CNN model. Similarly, the Inception structure in DeepSpectra can extract multi-scale feature information from bimodal signals, which also helps improve feature utilization and prediction performance compared to the single-scale convolution of simple CNN. For CNN-LSTM model, it is better than that of CNN model, CNN-ResNet model and DeepSpectra model, but CNN-MAM model is worse than that of CNN model, which demonstrates that the introduction of LSTM modlue can effectively capture the long-term and short-term sequential correlation features in the fused bimodal data, thereby improving the prediction ability of the model, while the simple combination of CNN and MAM module cannot fully exploit the multi-attention interaction of the extracted features, and even increases the difficulty of model feature learning, resulting in the decline of prediction performance. However, for DB-CNN-LSTM-MAM model, after combining the one-dimensional CNN processing NIR data, two-dimensional CNN processing muti-wavelength TR-PAS data, and further accessing LSTM and MAM modules, the prediction performance of the complete DB-CNN-LSTM-MAM model obtains the best results on all three SBIs. The reason may be that the incorporation of the MAM modules into the dual-branch CNN-LSTM model offers several distinct advantages for quantitative prediction of serum biochemical indicators. First, MAM enables the model to dynamically weigh the importance of different spectral features extracted by the dual-branch architecture from multi-wavelength time-resolved PA signals and NIR spectra. Unlike conventional CNN-LSTM models that may treat all temporal and spectral features equally, MAM allows the model to focus on the most informative features relevant to GLU, TG, and TC. Second, the parallel multi-head structure enhances the model’s ability to jointly attend to information from different representation subspaces at different positions, improving robustness against spectral noise and baseline shifts. Therefore, it can be known that the synergistic effect of each module in the model proposed in this work effectively integrates the bimodal spectral feature information, improves the feature extraction and utilization efficiency, and ultimately achieves better quantitative prediction results than the model missing any module. Consequently, the quantitative prediction performance of GLU, TG, and TC based on the DB-CNN-LSTM-MAM model proposed in this study is excellent.

At present, this research only focuses on the quantitative detection of three SBIs, i.e., GLU, TG and TC, so the universality for other SBIs or whole blood samples still needs further experimental verification. The core idea of the proposed algorithm framework, that is, combining step-by-step characteristic wavelength selection with weighted deep feature extraction, has certain general applicability. It can be extended to the detection of other SBIs after fine-tuning, but the specific performance still needs to be verified through a large number of actual sample experiments in the our future research.

### 5.2 Comparison between bimodal spectroscopy and unimodal spectroscopy

To validate the feasibility of bimodal spectroscopy, namely, TR-PAS combined with NIRS, the quantitative prediction performances of GLU, TG, and TC based on TR-PAS-NIRS were compared with those of two types of unimodal spectroscopies, specifically, unimodal NIRS and unimodal PAS. For unimodal NIRS, two DL models, the 1DCNN-LSTM model and the 1DCNN-LSTM with multi-head attention mechanism (1DCNN-LSTM-MAM) model, were employed, along with three ML models, including PLSR, SVR, and random forest (RF) [80]. Similar to the bimodal spectroscopy, prior to using the NIR spectral data for modeling, data augmentation, outlier removal, spectral preprocessing, and characteristic wavelength selection were conducted. For unimodal PAS, the augmented time-resolved PA signals of GLU, TG, and TC at different characteristic wavelengths were utilized for quantitative modeling using the 2DCNN model, the 2DCNN-LSTM model, and the 2DCNN-LSTM-MAM model, respectively. The root-mean-squared errors (RMSEC, and RMSEP) and coefficients of determination (R_c_^2^, and R_p_^2^) for both the training set and testing set were used as the evaluation indices for the quantitative performance of GLU, TG, and TC, which are presented in Table 2. From Table 2, it can be observed that, for unimodal NIRS, the prediction performances of GLU, TG, and TC based on the 1DCNN-LSTM-MAM model are superior to those of the 1DCNN-LSTM model and the other three ML models. The prediction results of GLU, TG, and TC in the testing set for unimodal NIRS based on the 1DCNN-LSTM-MAM model are presented in Fig. S2 (a)-(c) of Supplementary Material, respectively. For unimodal PAS, the prediction performances of GLU, TG, and TC based on the 2DCNN-LSTM-MAM model are better than those of the other two CNN models. The prediction results of GLU, TG, and TC in the testing set for unimodal PAS based on the 2DCNN-LSTM-MAM model are presented in Fig. S2(d)-(f) of Supplementary Material, respectively. However, from Table 2, it can be found that the prediction performances of GLU, TG, and TC for bimodal spectroscopy (TR-PAS**–**NIRS) based on the DB-CNN-LSTM-MAM model are all superior to those of unimodal NIRS and unimodal PAS.

**Table 2:** Comparative analysis of modeling between bimodal spectroscopy and unimodal spectroscopy for GLU, TG, and TC.

**SBI**	**Modality**	**Preprocessing Method**	**Model**	**Training set**		**Testing set**	
				**RMSE** **(mmol/L)**	**R**_**c**_^**2**^	**RMSEP** **(mmol/L)**	**R**_**p**_^**2**^
GLU	NIRS	SG + 1D + CARS(203)	PLSR SVR RF 1DCNN-LSTM 1DCNN-LSTM-MAM	1.413 1.517 1.232 0.963 0.843	0.683 0.613 0.757 0.861 0.901	1.531 1.742 1.315 1.135 0.899	0.651 0.563 0.731 0.831 0.881
	TR-PAS	NONE	2DCNN 2DCNN-LSTM 2DCNN-LSTM-MAM	1.996 1.535 0.965	0.514 0.690 0.875	2.191 1.641 1.132	0.504 0.666 0.854
	**TR-PAS–NIRS**	**SG + 1D + CARS (458)**	**DB-CNN-LSTM-MAM**	**0.376**	**0.989**	**0.9653**	**0.9296**
TG	NIRS	SG + 1D + CARS(304)	PLSR SVR RF 1DCNN-LSTM 1DCNN-LSTM-MAM	0.633 0.595 0.7656 0.690 0.493	0.758 0.807 0.783 0.831 0.932	0.753 0.615 0.793 0.755 0.556	0.694 0.735 0.776 0.803 0.893
	TR-PAS	NONE	2DCNN 2DCNN-LSTM 2DCNN-LSTM-MAM	0.715 0.663 0.601	0.752 0.841 0.836	0.794 0.694 0.659	0.718 0.813 0.803
	**TR-PAS–NIRS**	**SG + 1D + CARS (349)**	**DB-CNN-LSTM-MAM**	**0.280**	**0.977**	**0.3956**	**0.9575**
TC	NIRS	SG + 1D + CARS(266)	PLSR SVR RF 1DCNN-LSTM 1DCNN-LSTM-MAM	0.553 0.699 1.135 0.615 0.516	0.893 0.697 0.741 0.843 0.913	0.740 0.903 1.434 0.654 0.593	0.780 0.623 0.683 0.816 0.876
	TR-PAS	NONE	2DCNN 2DCNN-LSTM 2DCNN-LSTM-MAM	0.722 0.656 0.523	0.710 0.786 0.911	0.763 0.690 0.587	0.674 0.769 0.896
	**TR-PAS–NIRS**	**SG + 1D + CARS (457)**	**DB-CNN-LSTM-MAM**	**0.295**	**0.964**	**0.4443**	**0.9290**

### 5.3 Comparison between single-wavelength TR-PAS and multi-wavelength TR-PAS in biomodal spectroscopy

To verify the feasibility of NIRS combined with multi-wavelength TR-PAS, the prediction results of GLU, TG, and TC were compared between NIRS combined with multi-wavelength TR-PAS and NIRS combined with single-wavelength TR-PAS. For the single-wavelength TR-PAS branch, the time-resolved PA signals of serum samples at all single wavelength were selected from the multiple characteristic wavelengths of GLU, TG, and TC, respectively. The comparison results of GLU, TG, and TC based on the DB-CNN-LSTM-MAM model are presented in Table 3.

**Table 3:** Comparison results of predicting GLU, TG, and TC between NIRS with multi-wavelength TR-PAS and NIRS with single-wavelength TR-PAS.

SBI	Modality	Training set		Testing set	
		RMSEC (mmol/L)	R_c_^2^	RMSEP (mmol/L)	R_p_^2^
GLU	NIRS–single-wavelength TR-PAS (704 nm)	1.082	0.812	1.215	0.783
	NIRS–single-wavelength TR-PAS (720 nm)	0.936	0.865	1.013	0.845
	NIRS–single-wavelength TR-PAS (724 nm)	1.025	0.833	1.147	0.806
	NIRS–single-wavelength TR-PAS (734 nm)	0.971	0.858	1.069	0.827
	NIRS–single-wavelength TR-PAS (746 nm)	0.985	0.851	1.084	0.821
	NIRS–single-wavelength TR-PAS (750 nm)	1.104	0.805	1.208	0.790
	NIRS–single-wavelength TR-PAS (756 nm)	0.928	0.876	0.995	0.849
	NIRS–single-wavelength TR-PAS (758 nm)	1.046	0.826	1.162	0.798
	NIRS–single-wavelength TR-PAS (802 nm)	1.067	0.818	1.189	0.791
	NIRS–single-wavelength TR-PAS (812 nm)	0.950	0.870	1.020	0.840
	NIRS–single-wavelength TR-PAS (820 nm)	0.943	0.868	1.036	0.838
	NIRS–single-wavelength TR-PAS (834 nm)	1.115	0.798	1.224	0.776
	**NIRS–multi-wavelength TR-PAS**	**0.376**	**0.989**	**0.9653**	**0.9296**
TG	NIRS–single-wavelength TR-PAS (728 nm)	0.964	0.778	1.286	0.732
	NIRS–single-wavelength TR-PAS (748 nm)	0.919	0.802	1.207	0.761
	NIRS–single-wavelength TR-PAS (754 nm)	0.750	0.881	0.930	0.860
	NIRS–single-wavelength TR-PAS (798 nm)	1.031	0.739	1.379	0.691
	NIRS–single-wavelength TR-PAS (824 nm)	0.827	0.847	1.056	0.814
	NIRS–single-wavelength TR-PAS (840 nm)	0.865	0.831	1.118	0.793
	NIRS–single-wavelength TR-PAS (908 nm)	0.738	0.885	0.917	0.866
	NIRS–single-wavelength TR-PAS (940 nm)	0.726	0.889	0.904	0.871
	NIRS–single-wavelength TR-PAS (960 nm)	1.003	0.754	1.342	0.706
	NIRS–single-wavelength TR-PAS (970 nm)	0.779	0.868	0.978	0.842
	NIRS–single-wavelength TR-PAS (982 nm)	0.810	0.852	0.982	0.831
	NIRS–single-wavelength TR-PAS (1062 nm)	1.072	0.717	1.418	0.673
	NIRS–single-wavelength TR-PAS (1064 nm)	1.084	0.711	1.426	0.668
	**NIRS–multi-wavelength TR-PAS**	**0.280**	**0.977**	**0.3956**	**0.9575**
TC	NIRS–single-wavelength TR-PAS (720 nm)	0.927	0.839	1.115	0.792
	NIRS–single-wavelength TR-PAS (725 nm)	0.831	0.824	1.163	0.774
	NIRS–single-wavelength TR-PAS (818 nm)	0.863	0.870	1.003	0.830
	NIRS–single-wavelength TR-PAS (822 nm)	0.954	0.864	1.028	0.821
	NIRS–single-wavelength TR-PAS (882 nm)	0.824	0.875	0.962	0.838
	NIRS–single-wavelength TR-PAS (890 nm)	0.831	0.873	0.971	0.835
	NIRS–single-wavelength TR-PAS (934 nm)	1.016	0.787	1.272	0.733
	NIRS–single-wavelength TR-PAS (970 nm)	1.073	0.749	1.368	0.695
	NIRS–single-wavelength TR-PAS (982 nm)	1.114	0.721	1.435	0.668
	NIRS–single-wavelength TR-PAS (1060 nm)	1.152	0.693	1.481	0.642
	**NIRS–multi-wavelength TR-PAS**	**0.295**	**0.964**	**0.4443**	**0.9290**

As can be observed from Table 3, the wavelengths of best prediction performances of NIRS with single-wavelength TR-PAS for of GLU, TG, and TC are 756 nm, 940 nm, and 882 nm, respectively. However, for NIRS with multi-wavelength TR-PAS, its quantitative prediction performance of GLU, TG, and TC are all superior to those of NIRS with single-wavelength TR-PAS. This superiority is evidenced by lower RMSEC and RMSEP, as well as higher R_c_^2^ and R_p_^2^. Consequently, it can be shown that the approach of NIRS combined with multi-wavelength TR-PAS based on DL is available. However, since the characteristic wavelengths of GLU, TG, and TC are determined using the pure standards solution rather than the serum matrix or spiked serum samples, which may result in a limitation on the the lack of spike-recovery or matrix-effect validation. In the future work, this situation will be further considered.

### 5.4 Model validation of external independent samples

**Fig. 12:**
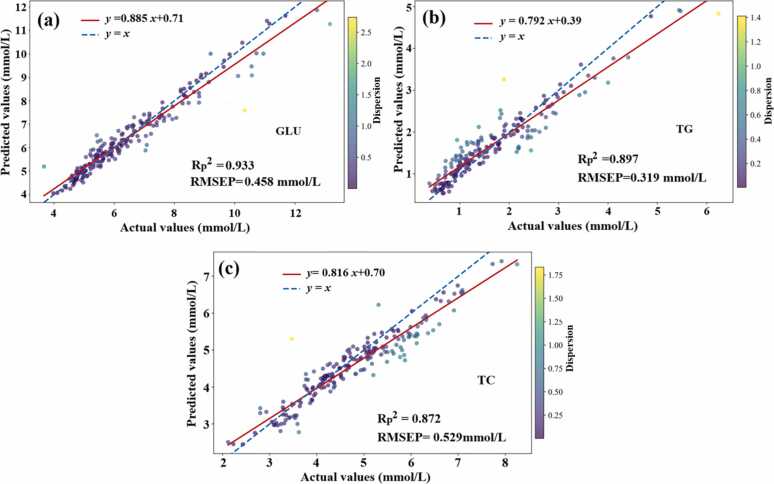
The predicted results of GLU (a), TG (b), and TC (c) for external independent testing set samples based on DB-CNN-LSTM-MAM model.

To verify the availability of established DB-CNN-LSTM-MAM model for the quantitative prediction performance of GLU, TG, and TC of external independent serum samples, this study designed to validate whether the model trained on the a batch of samples can still maintain effective predictive performance for samples collected in a different batch, which can better reflect the generalization ability of the model. That is, the external independent samples employed in this study specifically means that all samples in the external independent serum samples come from a different collection batch. In this work, the original 1084 serum samples were employed as the training set, and the other 200 serum samples were utilized as the testing set according to the batches. Moreover, there are no identical samples shared between the original training set and the external independent testing set. Based on the established DB-CNN-LSTM-MAM model, the predicted results of testing set samples for GLU, TG, and TC were obtained, which are shown in Fig. 12(a)-(c), respectively. For GLU, the RMSEC and R_c_^2^ of the training set are 0.433 mmol/L, and 0.941, respectively; the RMSEP and R_p_^2^ of testing set are 0.458 mmol/L, and 0.933, respectively. For TG, the RMSEC and R_c_^2^ of the training set are 0.234 mmol/L, and 0.920, respectively; the RMSEP and R_p_^2^ of testing set are 0.319 mmol/L, and 0.897, respectively. For TC, the RMSEC and R_c_^2^ of the training set are 0.499 mmol/L, and 0.888, respectively; the RMSEP and R_p_^2^ of testing set are 0.529 mmol/L, and 0.872, respectively.

From Fig. 12(a)-(c), it can be seen that the distribution of 200 external independent serum samples for GLU, TG, and TC is the same as those of the original serum samples, which conforms to the normal social distribution pattern of patients. Most of serum samples are located within the normal concentration range of GLU, TG, and TC, i.e., 3.9 ≤ GLU < 6.1 mmol/L, TG < 1.7 mmol/L, and 3.1 ≤ TC ≤ 5.2 mmol/L, followed by those within the slightly higher concentration range of GLU, TG, and TC, i.e., 6.1 ≤ GLU < 8.4 mmol/L, 1.7 ≤ TG ≤ 2.25 mmol/L, and 5.23 < TC ≤ 6.2 mmol/L. Moreover, from Fig. 12(a)-(c), it can be known that although the R_p_^2^ of GLU for external independent samples is close to that of the non-independent external samples (See Fig. 10a), the R_p_^2^ of TG and TC for external independent samples are slightly lower than those of the non-independent external samples (See Fig. 10(b), and c). This slight decrease in prediction performance of TG and TC may be attributed to the following reasons: 1) there are the certain differences among batches of biochemical reagents or the errors in the collected near-infrared spectral data and muliti-wavelength TR-PA signals caused by the environmental fluctuations and samples temperature deviation. 2) the DB-CNN-LSTM-MAM model has not come into contact with any samples from the external independent batch during the training process, leading to a certain degree of prediction deviation. 3) the Spearman correlation coefficient (*ρ*) between the distance of each external independent samples from the center of the training set and its predicted residual was calculated. The *ρ* for TG and TCof the external independent serum samples are 0.378 and 0.224 (p < 0.001). As we all know, the farther the external samples are from the distribution of training data, i.e., the larger the *ρ*, the greater the prediction error. This indicates that the data shift of external independent samples may be the main factor leading to the degradation of TG and TC prediction performance. 4) comparing the regression equations in Fig. 10(b-c) and Fig. 12 (b-c), it can be observed that the the intercepts are greatly increased from the internal serum samples to the external independent samples, which may be demonstrated that the reagent calibration drift or baseline noise interference has systematically elevated the predicted results of all serum samples. In addition, to verify the acceptness of prediction deviation for TG and TC in the practical clinical application scenarios, the mean deviation percentages are computed between the prediction value and the actual value of TG, and TC, the mean deviation percentages of TG and TC are 10.96%, and 7.3%, respectively, which are less than the clinically accepted error thresholds, i.e., 15% and 9% for TG and TC [81]. Therefore, the overall prediction error remains within a reasonable range, and the model still maintains good generalization ability for samples from different batches, which confirms that the proposed NIRS–multi-wavelength TR-PAS method combined with the DB-CNN-LSTM-MAM model has good practical applicability for quantitative detection of SBIs.

## 6 Conclusion

In this work, a bimodal spectroscopy method combining multi-wavelength TR-PAS with NIRS, integrated with DL, has been proposed for the quantitative detection of three human SBIs, including GLU, TG, and TC. To achieve this goal, 12, 13, and 10 characteristic wavelengths are determined for the energy-corrected PA peak-to-peak value spectra of GLU, TG, and TC standard solutions, respectively, by integrating the CARS, BOSS, and SPA algorithms. Energy-corrected time-resolved PA signals at the aforementioned 35 characteristic wavelengths, along with the NIR spectra of serum samples, were collected experimentally. The preprocessed NIR spectra and multi-wavelength time-resolved PA signals were then used to establish quantitative models for GLU, TG, and TC. The main conclusions are summarized as follows:

(1) To fully mine the information representing SBIs concentration from both NIR spectra and time-resolved PA signals, a dual-branch DL framework, namely the DB-CNN-LSTM-MAM model, is constructed to quantitatively predict GLU, TG, and TC, respectively. Using this model, the RMSEP for the testing set regarding the three SBIs (GLU, TG, and TC) are 0.9653, 0.3956, and 0.4443 mmol/L, respectively, and the coefficients of determination (R_p_²) are all above 0.9. The comparative results with other DL models demonstrate that the DB-CNN-LSTM-MAM model exhibits excellent quantitative prediction performance.
(2) The 10-fold cross validation results of the trainging set for GLU, TG, and TC, as well as the model validation results for the external independent serum samples were all performed, which fully verify the availability and generalization of quantitative predicting GLU, TG, and TC based on established DB-CNN-LSMT-MAM model.
(3) Compared with unimodal spectroscopy, the bimodal spectroscopy fusion method (i.e., TR-PAS–NIRS) achieves better quantitative prediction of SBIs, thereby verifying the feasibility of combining bimodal spectroscopy with DL.
(4) Compared with single-wavelength TR-PAS**–**NIRS, the prediction performances for GLU, TG, and TC based on multi-wavelength TR-PAS**–**NIRS are superior, which fully validated the effectiveness of the multi-wavelength TR-PAS**–**NIRS, and provides significant technical supports for the quantitative measurement of SBIs.

## CRediT authorship contribution statement

**Zhong Ren:** Writing – review & editing, Writing – original draft, Supervision, Project administration, Methodology, Funding acquisition, Conceptualization. **Chaojun Chen:** Software, Investigation, Data curation. **Gaoqiang Liang:** Writing – review & editing, Validation, Software, Methodology. **Haibin Zhang:** Writing – review & editing, Visualization, Formal analysis, Data curation. **Weinan Shi:** Resources, Investigation, Formal analysis. **Jia Zhang:** Writing – review & editing, Validation, Investigation, Formal analysis. **Guohui Xiao:** Validation, Resources, Investigation. **Xiaoyu Zhu:** Writing – review & editing, Validation, Formal analysis. **Wenyan Nie:** Validation, Formal analysis, Data curation.

## Funding

This work was supported by the 10.13039/501100001809National Natural Science Foundation of China (62165006), Jiangxi Province Ganpo Juncai Support PlanHigh level and High skilled Leading Talent Training Project (2024–069), Nanchang City Key Laboratory of Optic-electronic Detection and Information Processing (2019-NCZDSY−008), Jiangxi Province Natural and Science Fund (20224ACB202004, 20242BAB20065, 20262BAC260001), Key Research and Development Program Project of Jiangxi Province (20243BBI91011), and Jiangxi Provincial Science and Technology Project of Education Department (GJJ2401207).

## Declaration of Competing Interest

The authors declare that there are no conflicts of interest.

## Data Availability

Data will be made available on request.

## Footnotes

Appendix A Supplementary data associated with this article can be found in the online version at doi:10.1016/j.pacs.2026.100858.
