# Supplementary material for: Bimodal spectroscopy integrating multi-wavelength time-resolved photoacoustic spectroscopy and near-infrared spectroscopy with deep learning for quantitative detection of serum biochemical indicators
Source: Photoacoustics. 2026 Jul 10;51:100858. doi: 10.1016/j.pacs.2026.100858 (PMC13400291; doi:10.1016/j.pacs.2026.100858)
Supplement: Supplementary file 1 — Supplementary material [file mmc1.docx]

**Table S1.** Comparison of spectra preprocessing methods for GLU, TG, and TC

| **SBIs** | **Preprocessing method** | **PCs*** | **Calibration set** | | **Prediction set** | |
| --- | --- | --- | --- | --- | --- | --- |
|  |  |  | **RMSEC (mmol/L)** | **R_c_^2^** | **RMSEP**  **(mmol/L)** | **R_p_^2^** |
| GLU | FD | 13 | 1.661 | 0.561 | 1.802 | 0.413 |
|  | SG | 20 | 1.683 | 0.546 | 1.735 | 0.510 |
|  | MSC | 19 | 1.814 | 0.473 | 1.842 | 0.453 |
|  | MAF | 11 | 1.983 | 0.415 | 2.031 | 0.404 |
|  | SNV | 18 | 1.913 | 0.445 | 1.998 | 0.431 |
|  | **SG+FD** | **19** | **1.413** | **0.683** | **1.531** | **0.651** |
|  | MSC+FD | 17 | 1.612 | 0.583 | 1.684 | 0.571 |
|  | MAF+FD | 14 | 2.054 | 0.433 | 2.137 | 0.415 |
|  | SNV+FD | 9 | 1.731 | 0.434 | 1.877 | 0.402 |
|  | SNV+SG | 16 | 1.831 | 0.463 | 1.973 | 0.421 |
|  | MSC+SG | 20 | 1.697 | 0.536 | 1.758 | 0.467 |
| TG | FD | 8 | 0.911 | 0.513 | 1.004 | 0.431 |
|  | SG | 23 | 0.892 | 0.541 | 0.924 | 0.511 |
|  | MSC | 13 | 1.053 | 0.452 | 1.091 | 0.442 |
|  | MAF | 11 | 1.041 | 0.462 | 1.104 | 0.456 |
|  | SNV | 15 | 1.011 | 0.457 | 1.091 | 0.446 |
|  | **SG+FD** | **20** | **0.593** | **0.758** | **0.753** | **0.694** |
|  | MSC+FD | 29 | 0.791 | 0.643 | 0.841 | 0.564 |
|  | MAF+FD | 28 | 0.955 | 0.476 | 1.017 | 0.421 |
|  | SNV+FD | 5 | 0.963 | 0.471 | 1.132 | 0.454 |
|  | SNV+SG | 29 | 0.873 | 0.562 | 0.921 | 0.514 |
|  | MSC+SG | 6 | 0.936 | 0.497 | 1.101 | 0.452 |
| TC | FD | 22 | 1.127 | 0.570 | 1.156 | 0.466 |
|  | SG | 16 | 0.723 | 0.640 | 0.776 | 0.546 |
|  | MSC | 24 | 1.122 | 0.569 | 1.335 | 0.541 |
|  | MAF | 19 | 1.394 | 0.541 | 1.452 | 0.492 |
|  | SNV | 13 | 1.311 | 0.499 | 1.405 | 0.438 |
|  | **SG+FD** | **18** | **0.553** | **0.893** | **0.740** | **0.780** |
|  | MSC+FD | 17 | 0.792 | 0.612 | 0.816 | 0.576 |
|  | MAF+FD | 7 | 1.320 | 0.513 | 1.513 | 0.473 |
|  | SNV+FD | 11 | 0.713 | 0.664 | 0.851 | 0.563 |
|  | SNV+SG | 15 | 1.159 | 0.530 | 1.231 | 0.513 |
|  | MSC+SG | 21 | 0.973 | 0.563 | 1.103 | 0.543 |

PCs^*^: principal components.
