# Supplementary material for: Bimodal spectroscopy integrating multi-wavelength time-resolved photoacoustic spectroscopy and near-infrared spectroscopy with deep learning for quantitative detection of serum biochemical indicators
Source: Photoacoustics. 2026 Jul 10;51:100858. doi: 10.1016/j.pacs.2026.100858 (PMC13400291; doi:10.1016/j.pacs.2026.100858)
Supplement: Supplementary file 2 — Supplementary material [file mmc2.docx]

**Table S2.** Structure and parameters of DB-CNN-LSTM-MAM model for predicting GLU, TG, and TC

| SBIs | Branch | Network Layer | Kernal size | Stride | Padding | Channels |
| --- | --- | --- | --- | --- | --- | --- |
| GLU | NIRS | 1D Conv1  1D BN  Maxpooling  1D Conv2  1D BN  Maxpooling  1D Conv3  1D BN | 3×1  -  3×1  5×1  -  3×1  7×1  - | 1  -  1  1  -  1  2  - | 0  -  0  1  -  0  0  - | 8  8  -  16  16  -  32  32 |
|  | PAS | 2D Conv1  2D BN  2D Maxpooling  2D Conv2  2D BN  2D Maxpooling  2D Conv3  2D BN | 3×3  -  3×3  5×5  -  3×3  3×3  - | 1  -  1  1  -  1  2  - | ‘same’  -  ‘same’  ‘valid’  -  ‘same’  ‘same’  - | 32  32  -  64  64  -  128  128 |
|  | LSTM module | Hidden size: 64 | Number of layers: 3 | - | - | - |
|  | MAM module | - | - | Attention size: 64 | Number of heads:4 | FC: 64 |
| TG | NIRS | 1D Conv1  1D BN  Maxpooling  1D Conv2  1D BN  Maxpooling  1D Conv3  1D BN | 3×1  -  3×1  5×1  -  3×1  3×1  - | 1  -  1  1  -  1  2  - | 0  -  0  1  -  0  0  - | 8  8  -  16  16  -  32  32 |
|  | PAS | 2D Conv1  2D BN  2D Maxpooling  2D Conv2  2D BN  2D Maxpooling  2D Conv3  2D BN | 3×3  -  5×5  3×3  -  3×3  3×3  - | 1  -  1  1  -  1  2  - | ‘same’  -  ‘same’  ‘valid’  -  ‘valid’  ‘valid’  - | 16  16  -  32  32  -  64  64 |
|  | LSTM module | Hidden size: 128 | Number of layers:2 | - | - | - |
|  | MAM module | - | - | Attention size:128 | Number of heads: 2 | FC: 128 |
| TC | NIRS | 1D Conv1  1D BN  Maxpooling  1D Conv2  1D BN  Maxpooling  1D Conv3  1D BN | 3×1  -  3×1  5×1  -  3×1  3×1  - | 1  -  1  1  -  1  2  - | 0  -  0  1  -  0  0  - | 16  16  -  32  32  -  64  64 |
|  | PAS | 2D Conv1  2D BN  2D Maxpooling  2D Conv2  2D BN  2D Maxpooling  2D Conv3  2D BN | 3×3  -  3×3  5×5  -  3×3  3×3  - | 1  -  1  1  -  1  2  - | ‘valid’  -  ‘same’  ‘same’  -  ‘same’  ‘same’  - | 16  16  -  32  32  -  64  64 |
|  | LSTM module | Hidden size: 128 | Number of layers: 2 | - | - | - |
|  | MAM module | - | - | Attention size: 128 | Number of heads: 2 | FC: 128 |
