# Supplementary material for: Bimodal spectroscopy integrating multi-wavelength time-resolved photoacoustic spectroscopy and near-infrared spectroscopy with deep learning for quantitative detection of serum biochemical indicators
Source: Photoacoustics. 2026 Jul 10;51:100858. doi: 10.1016/j.pacs.2026.100858 (PMC13400291; doi:10.1016/j.pacs.2026.100858)
Supplement: Supplementary file 3 — Supplementary material [file mmc3.docx]

**Table S3.** The quantitative prediction results and statistic information of training set and testing set for GLU, TG, and TC at five repeated training times based on BD-CNN-LSTM-MAM model.

| SBI | Parameters | Random Seed | Training set | | Testing set | | R_p_^2^ | | RMSEP (mmol/L) | | 10-fold cross validation | |
| --- | --- | --- | --- | --- | --- | --- | --- | --- | --- | --- | --- | --- |
|  |  |  | R_c_^2^ | RMSEC  (mmol/L) | R_p_^2^ | RMSEP  (mmol/L) | Mean ± SD* | BI* | Mean ±  SD | BI | Mean ± SD of R_cv_^2^ | Mean ± SD of RMSECV (mmol/L) |
| GLU | LR*=0.001, lambda_L_2_*=0.001,  Batch_size*=116, epoch=100,  TBatch_size*=240,  hid*=128, num_layers*=2 | seed0 | 0.9934 | 0.2989 | 0.9313 | 0.9513 | 0.9296 ± 0.0026 | [0.9276, 0.9315] | 0.9653 ± 0.0177 | [0.9522, 0.9790] | 0.927±0.016 | 0.993±0.095 |
|  |  | seed1 | 0.9897 | 0.3733 | 0.9291 | 0.9671 |  |  |  |  |  |  |
|  |  | seed42 | 0.9824 | 0.4857 | 0.9329 | 0.9442 |  |  |  |  |  |  |
|  |  | seed123 | 0.9905 | 0.357 | 0.9283 | 0.977 |  |  |  |  |  |  |
|  |  | seed2025 | 0.9899 | 0.3667 | 0.9262 | 0.9869 |  |  |  |  |  |  |
| TG | LR=0.001, lambda_L_2_=0.001  Batch_size=16, epoch=100, TBatch_size=240, hid=128, num_layers=2 | seed0 | 0.9791 | 0.2746 | 0.9558 | 0.4068 | 0.9575 ± 0.0015 | [0.9565, 0.9586] | 0.3956 ± 0.0098 | [0.3873, 0.4024] | 0.947±0.013 | 0.424±0.02 |
|  |  | seed1 | 0.974 | 0.2938 | 0.9581 | 0.3994 |  |  |  |  |  |  |
|  |  | seed42 | 0.9748 | 0.2936 | 0.9573 | 0.3946 |  |  |  |  |  |  |
|  |  | seed123 | 0.9784 | 0.2642 | 0.9568 | 0.3972 |  |  |  |  |  |  |
|  |  | seed2025 | 0.9763 | 0.2727 | 0.9597 | 0.380 |  |  |  |  |  |  |
| TC | LR=0.001, lambda_L_2_=0.001,  Batch_size=16, epoch=100, TBatch_size=240 hid=128, num_layers=2 | seed0 | 0.9416 | 0.3819 | 0.9254 | 0.4522 | 0.929 ± 0.0023 | [0.9269, 0.9305] | 0.4443 ± 0.0063 | [0.4395, 0.4497] | 0.914±0.019 | 0.450±0.048 |
|  |  | seed1 | 0.9794 | 0.2322 | 0.9308 | 0.4388 |  |  |  |  |  |  |
|  |  | seed42 | 0.9575 | 0.3201 | 0.9303 | 0.4385 |  |  |  |  |  |  |
|  |  | seed123 | 0.9639 | 0.2984 | 0.9304 | 0.4425 |  |  |  |  |  |  |
|  |  | seed2025 | 0.976 | 0.2444 | 0.928 | 0.4497 |  |  |  |  |  |  |
| SD*: standard deviation; BI*: bootstrap intervals; lambda_L_2_*: super-parameter of L_2_ regularization; LR*: learning rate; Batch_size*: batch size of training set; TBatch_size*:batch size of testing set; hid*: hidden size of LSTM module; num_layers*: number of hidden layers for LSTM module. | | | | | | | | | | | | |
