# Supplementary figures and images for: Bimodal spectroscopy integrating multi-wavelength time-resolved photoacoustic spectroscopy and near-infrared spectroscopy with deep learning for quantitative detection of serum biochemical indicators
Source: Photoacoustics. 2026 Jul 10;51:100858. doi: 10.1016/j.pacs.2026.100858 (PMC13400291; doi:10.1016/j.pacs.2026.100858)

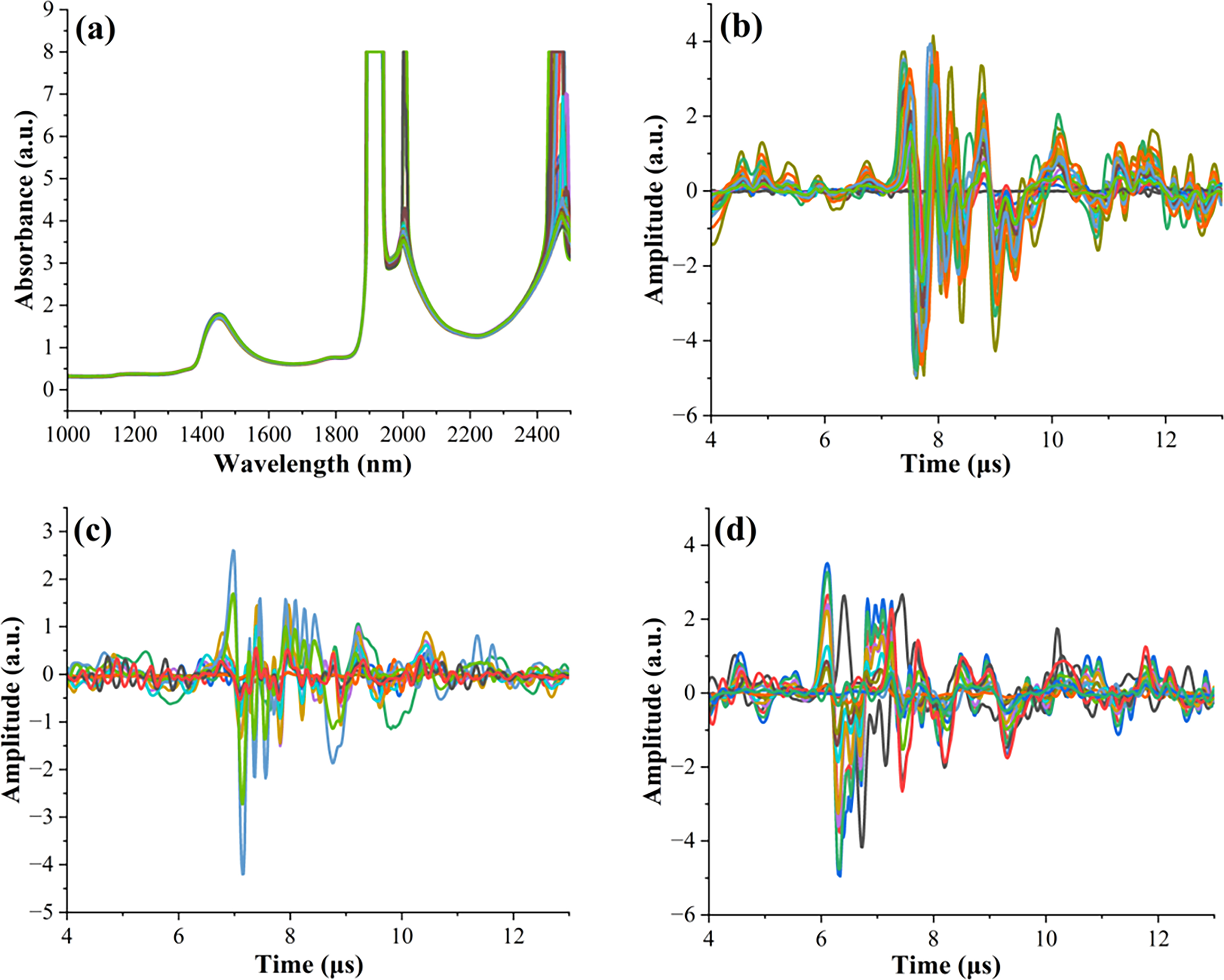

Supplement: Supplementary file 4 — Supplementary material [file mmc4.jpg]

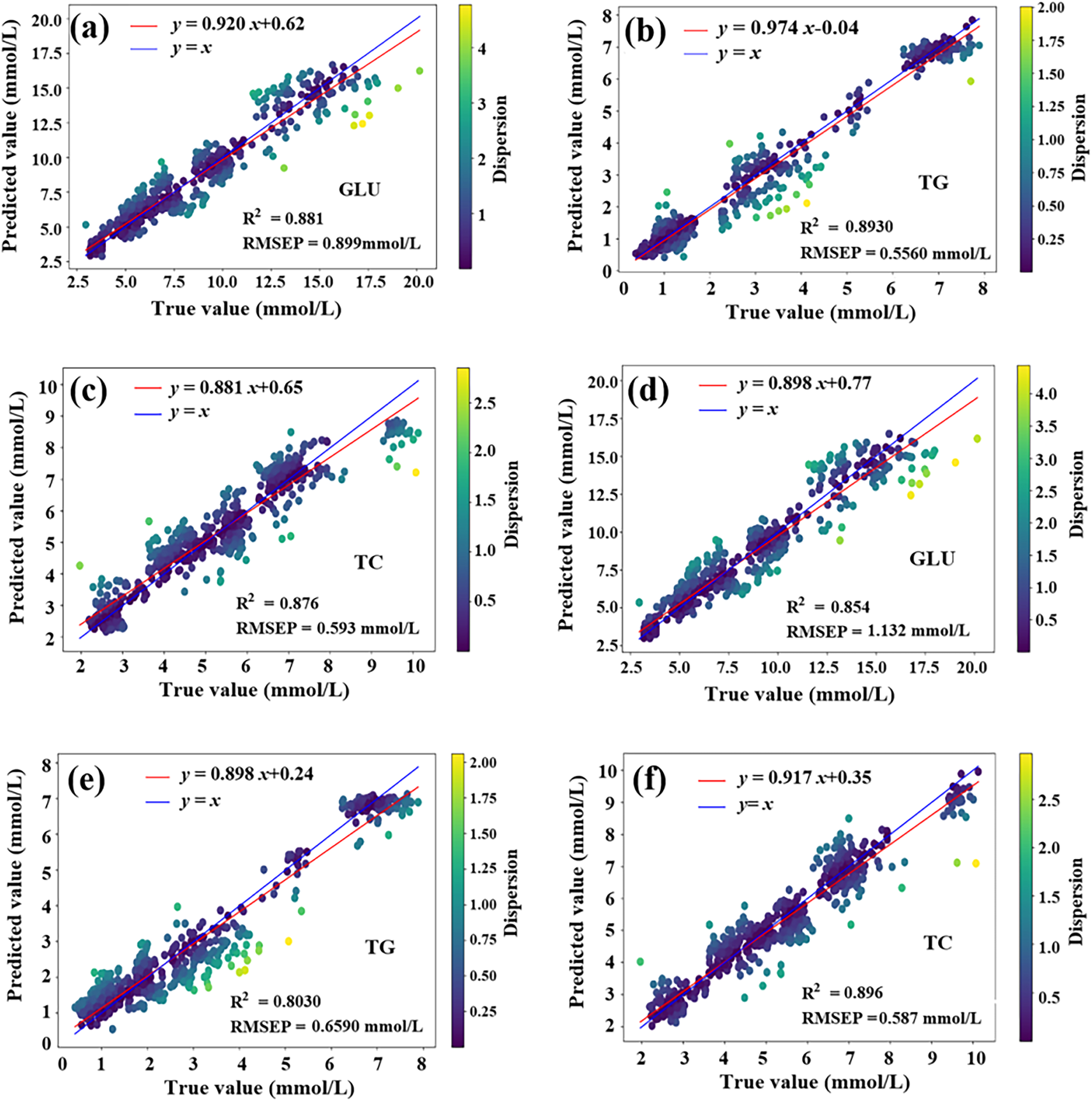

Supplement: Supplementary file 5 — Supplementary material [file mmc5.jpg]
